# Supplementary material for: Unsupervised detection and fitness estimation of emerging SARS-CoV-2 variants: Application to wastewater samples (ANRS0160)
Source: PLoS Comput Biol. 2025 Dec 3;21(12):e1013749. doi: 10.1371/journal.pcbi.1013749 (PMC12694877; doi:10.1371/journal.pcbi.1013749)
Supplement: S1 Text — (PDF) [file pcbi.1013749.s001.pdf]

Supporting Information S1 Text

Statistical method

Clustering and parameter estimation

We use the expectation-maximization (EM) algorithm for clustering and parameter estimation. Let $\theta = (\pi, \mu, s, \alpha, \beta)$ be the set of parameters, we perform maximization steps of the EM algorithm with the Lagrange multipliers for updating π, maximum likelihood estimator with the `glm` function of the `stats R package` for updating μ and s and the `vglm` function of the `VGAM R` package for updating α and β. We use posterior group assignment, that is, for $k \in \{0, \ldots, K\}, \mathbb{P}(Z_i = k \,|\, X_i = x_i; \theta)$ where $(x_i) = (x_{i,t})_{t=0,\ldots,T}$ denotes the observed vector of mutation counts, as weights in generalized linear models. The expectation steps are simply given by

$$\mathbb{P}(Z_i = k, k \neq 0 \,|\, X_i = x_i; \theta) \propto \pi_k \prod_{t \in \mathcal{T}} f_{i,t,k}(x_{i,t})$$

where $f_{i,t,k}$ is the density of the binomial distribution of parameters $d_{i,t}$ and $\text{Logistic}(\mu_k + s_k t)$ for non-neutral groups and by

$$\mathbb{P}(Z_i = 0 \,|\, X_i = x_i; \theta) \propto \pi_0 \prod_{t \in \mathcal{T}} \binom{d_{i,t}}{x_{i,t}} \frac{\prod_{a=0}^{(\sum_{t \in \mathcal{T}} x_{i,t})-1} (\alpha + a) \prod_{b=0}^{(\sum_{t \in \mathcal{T}} d_{i,t} - x_{i,t})-1} (\beta + b)}{\prod_{c=0}^{(\sum_{t \in \mathcal{T}} d_{i,t})-1} (\alpha + \beta + c)}$$

for the neutral group, using the fact that the beta distribution is a conjugate prior for the binomial distribution. The proof of that latter equation is provided below:

We have

$$\begin{aligned} \mathbb{P}(Z_i = 0 | X_i = x_i, \theta) &\propto \pi_0 \int_0^1 \prod_{t=0}^{T} \mathbb{P}(X_{i,t} = x_{i,t} | u, Z_i = 0; \theta) \mathbb{P}(u|\theta)\; du \\ &\propto \pi_0 \int_0^1 \prod_{t=0}^{T} \binom{d_{i,t}}{x_{i,t}} u^{x_{i,t}} (1-u)^{d_{i,t} - x_{i,t}} \mathbb{P}(u|\theta)\; du \end{aligned}$$

where we recall that $\mathbb{P}(Z_i = 0|\theta) = \pi_0$ and $u \sim \text{Beta}(\alpha, \beta)$, $\alpha, \beta > 0$, where Beta is the beta distribution. Returning to the definition of the beta distribution, we have

$$\mathbb{P}(u|\theta) = C_{\alpha,\beta}\, u^{\alpha-1} (1-u)^{\beta-1} \quad \text{where} \quad C_{\alpha,\beta} = \left(\int_0^1 u^{\alpha-1} (1-u)^{\beta-1}\; du \right)^{-1} = (\mathrm{B}(\alpha,\beta))^{-1}$$

where B is the beta function.

Let $A = \int_0^1 \prod_{t=0}^{T} \mathbb{P}(X_{i,t} = x_{i,t} | u, Z_i = 0; \theta) \mathbb{P}(u|\theta)\; du$, we therefore have

$$\begin{aligned} A &= C_{\alpha,\beta} \prod_{t=0}^{T} \binom{d_{i,t}}{x_{i,t}} \int_0^1 u^{\sum_{t=0}^{T} x_{i,t} + \alpha - 1} (1-u)^{\sum_{t=0}^{T} d_{i,t} - x_{i,t} + \beta - 1}\; du \\ A &= C_{\alpha,\beta} \prod_{t=0}^{T} \binom{d_{i,t}}{x_{i,t}} \mathrm{B}\left(\sum_{t=0}^{T} x_{i,t} + \alpha, \sum_{t=0}^{T} d_{i,t} - x_{i,t} + \beta \right). \end{aligned}$$

Denoting Γ, the gamma function, we recall that $\mathrm{B}(\alpha,\beta) = \frac{\Gamma(\alpha)\Gamma(\beta)}{\Gamma(\alpha+\beta)}$ and we obtain

$$A = \prod_{t=0}^{T} \binom{d_{i,t}}{xi,t} \frac{\Gamma(\sum_{t=0}^{T} x_{i,t} + \alpha)\Gamma(\sum_{t=0}^{T} d_{i,t} - x_{i,t} + \beta)}{\Gamma(\sum_{t=0}^{T} d_{i,t} + \alpha + \beta)} \frac{\Gamma(\alpha+\beta)}{\Gamma(\alpha)\Gamma(\beta)}.$$

We notice that

$$\begin{aligned} \frac{\Gamma(\sum_{t=0}^{T} x_{i,t} + \alpha)}{\Gamma(\alpha)} &= \frac{1}{\cancel{\Gamma(\alpha)}} \left(\sum_{t=0}^{T} x_{i,t} + \alpha - 1\right) \left(\sum_{t=0}^{T} x_{i,t} + \alpha - 2\right) \dots (\alpha+1)\, \alpha \cancel{\Gamma(\alpha)} \\ &= \prod_{a=0}^{\sum_{t=0}^{T} x_{i,t} - 1} (\alpha + a). \end{aligned}$$

Similarly we have

$$\frac{\Gamma(\sum_{t=0}^{T} d_{i,t} - x_{i,t} + \beta)}{\Gamma(\beta)} = \prod_{b=0}^{\sum_{t=0}^{T} d_{i,t} - x_{i,t} - 1} (\beta + b)$$

and

$$\frac{\Gamma(\sum_{t=0}^{T} d_{i,t} + \alpha + \beta)}{\Gamma(\alpha+\beta)} = \prod_{c=0}^{\sum_{t=0}^{T} d_{i,t} - 1} (\alpha + \beta + c)$$

which concludes the proof.

Finally, confidence intervals are empirically computed with the observed Fisher information matrix using `numDeriv` `R` package.

EM initialization

We faced a commonly encountered issue when using the EM algorithm, that is its high sensitivity to initialization. The EM algorithm is usually initialized with a chosen and empirically tested number of random initial values and results (estimates) associated to best log-likelihood are kept. One can either choose initial values for the parameter and starts with the Expectation step or initial values for latent variables (group assignment in our case) and starts with the Maximization step of the EM algorithm. In order to reduce time complexity, it is usual to solely perform a limited number of iterations for each initial value tested and results associated to the best (or a collection of some of the best) log-likelihood after these few iterations are used to initialize the algorithm and run it until convergence. A fully random proposal for initial values often prove to be insufficient, in particular when applied to real datasets (unless trying an unreasonable amount of initial values) and a usual option to overcome that issue is to propose data-driven initial values. We tested the following strategy to propose data-driven initial values for posterior group assignment, which proved to be efficient on various simulated and WWTP data from France and Switzerland. In this paragraph, our model composed of one neutral group and K non-neutral groups is denoted $\mathcal{M}_K^0$ and an alternative model composed of $K+1$ groups, all associated to fixed intercept and selection coefficient (equivalent to model $\mathcal{M}_K^0$ where the neutral group follows the same distribution as non-neutral groups with its own fixed intercept and selection coefficient) is denoted $\mathcal{M}_{K+1}$. Note that in the latter model, there is no random parameter and consequently there is no added variance for binomial distributions of mutation counts of any group. In such manner its captures less variability but it is more stable and much faster to fit. Models $\mathcal{M}_{K+2}$ and $\mathcal{M}_{K+3}$ are used to leverage data in order to propose,

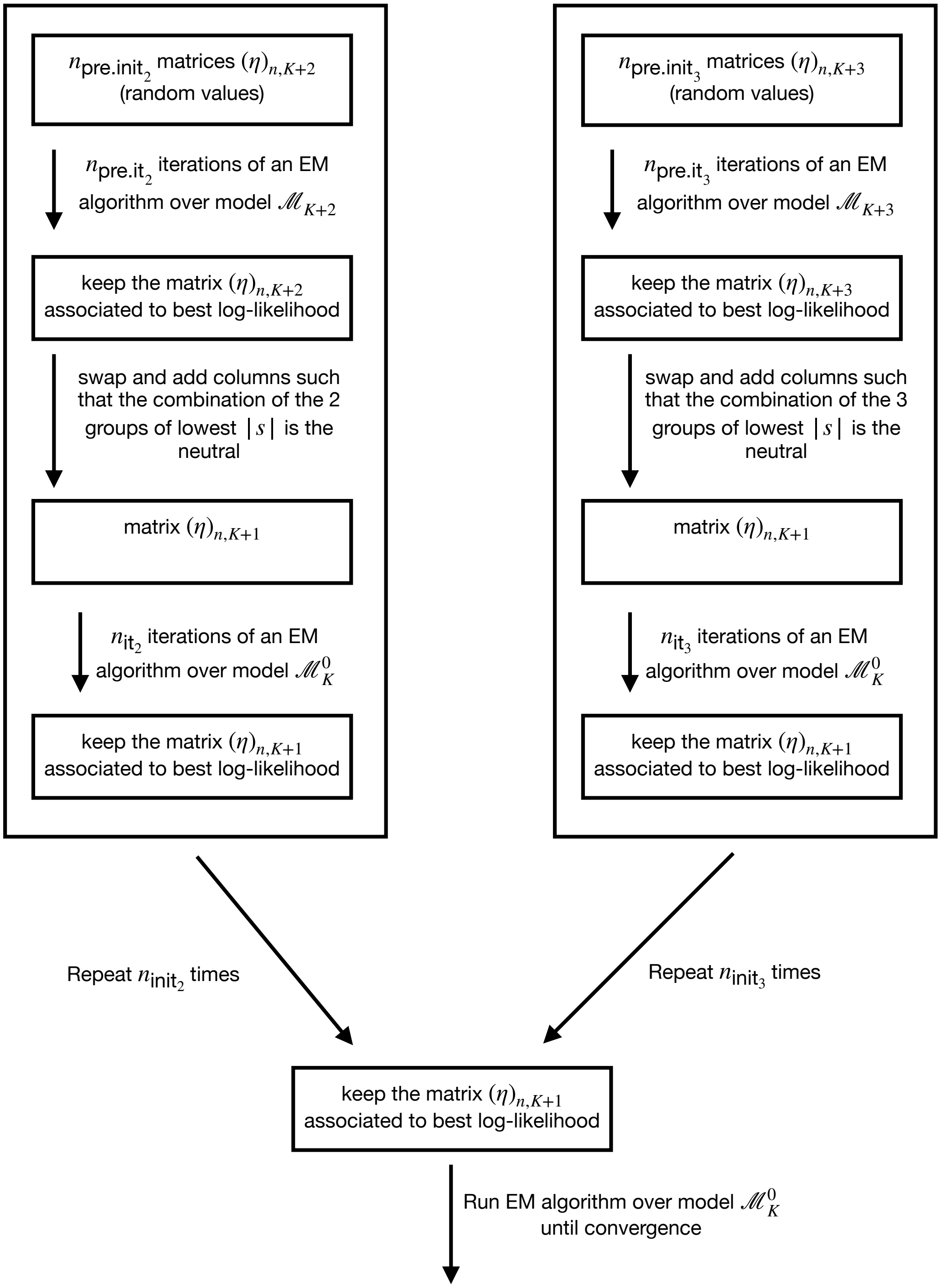

Fig S1. Flowchart of the initialization and running process where n is the number of mutations, $(\eta)_{n,K}$ is a $n \times K$ matrix of posterior group assignment for a model composed of a total of K groups, $\mathcal{M}_K^0$ denotes our model composed of 1 neutral group and K non-neutral groups, $\mathcal{M}_{K+2}$ (respectively $\mathcal{M}_{K+3}$) denotes a model composed of $K+2$ (respectively $K+3$) non-neutral groups and no neutral group.

$n_{\text{init}_2} + n_{\text{init}_3}$ data-driven initial values for the EM algorithm applied over our model $\mathcal{M}_K^0$. For a better comprehension, a flowchart of the process is represented in Fig S1 of the current supporting information file.

The n_{init_2} (respectively n_{init_3}) data-driven initial values computed with model $\mathcal{M}_{K+2}$ (respectively $\mathcal{M}_{K+3}$) are obtained by running n_{init_2} (respectively n_{init_3}) times the following procedure, where we denote by $(\eta)_{n,K}$, a $n \times K$ matrix of posterior group assignment in a model composed of a total of K groups: For $k \in \{2, 3\}$, we randomly sample $n_{\text{pre.init}_k}$ matrices $(\eta)_{n,K+k}$ as initial values of an EM algorithm over model $\mathcal{M}_{K+k}$ and run $n_{\text{pre.it}_k}$ iterations. The matrix $(\eta)_{n,K+k}$ associated to the best log-likelihood after the $n_{\text{pre.it}_k}$ iterations is used as initial values for our model $\mathcal{M}_K^0$, setting the k groups associated to lowest absolute values for selection coefficient estimates as the neutral group. For reducing time complexity, we solely run n_{it_k} iterations and keep, among the $\sum_{k=2}^{3} n_{\text{init}_k}$ matrices $(\eta)_{n,K+1}$, the one associated to best log-likelihood for pursuing iterations until convergence of the algorithm.

We noticed in particular that, for $k \in \{2, 3\}$, $n_{\text{pre.init}_k}$ and $n_{\text{pre.it}_k}$ should be kept low as the maximum likelihood estimator for a model with no neutral group is not necessarily the best start for a model with a neutral group. Moreover, the number of initial values to test $(n_{\text{init}_2} + n_{\text{init}_3})$ grows with K. Through our experience on several simulated and WWTP datasets, we suggest to set $(n_{\text{pre.init}_k})_{k\in\{2,3\}} = (5, 5)$, $(n_{\text{pre.it}_k})_{k\in\{2,3\}} = (5, 5)$, $(n_{\text{init}_k})_{k\in\{2,3\}} = (10, 10)$ and $(n_{\text{it}_k})_{k\in\{2,3\}} = (5, 5)$ for $K < 3$ and either rise $(n_{\text{init}_k})_{k\in\{2,3\}}$ to $(40, 40)$ or run the process 4 times for $K \geq 3$. We also recommend to rise $(n_{\text{it}_k})_{k\in\{2,3\}}$ to $(30, 30)$ in case of (rarely encountered) unstable results with lower values. These values should however be adapted to the context and datasets analyzed.

Algorithmic complexity

The algorithmic complexity in time for one run tested over several datasets of different sizes are provided in Table S1 of the current supporting information file for two different values of K with default number of initial values, that is, $(n_{\text{pre.init}_k})_{k\in\{2,3\}} = (n_{\text{pre.it}_k})_{k\in\{2,3\}} = (n_{\text{it}_k})_{k\in\{2,3\}} = (5, 5)$ and $(n_{\text{init}_k})_{k\in\{2,3\}} = (10, 10)$. These results were computed on an average laptop. One can run the algorithm over several different values of K and/or perform several runs using parallel computation with no increased complexity in time. The difference between the first and the second columns lies in the preparation of the datasets with different variant callers and different frequency thresholds applied, leading to different number of mutations kept. Nantes (respectively Lausanne and Zurich) datasets are those presented in the work of [1] (respectively in the work of [2] over a limited number of time points at the end of December) of the reference section in the current supporting information file. The different read depths ranges are explained by the different sequencing technologies used by the authors (Nanopore for Nantes versus Illumina for Lausanne and Zurich). We can notice that, along with the number of mutations and number of time points, read depths strongly drive the algorithmic complexity. For a computational boost, one can sample a collection of time points and/or divide both mutation counts and read depths data by the same quantity, in particular during initialization. Applying for instance that latter trick, for initialization and run, to Lausanne (and respectively Zurich) dataset using 10 (respectively 40) as denominator dropped the complexity below ten seconds for both datasets. In most cases, the average complexity of the algorithm (without the aforementioned tricks) ranges between few seconds and few minutes (up to 10 minutes), except when n is in the order of thousands and number of time points above ten. However, applying thresholds and alternative options to reduce the size of datasets is strongly encouraged to remove mutations of near zero frequency through time and gain

in statistical power.

Table S1. Time complexity.

	Nantes (2 WWTP)			Lausanne	Zurich
n	3186 to 3744	155 to 157	30 to 37	20	39
$m+1$	12 to 17	12 to 17	2	4	4
d quantiles 25%	123 to 128	33 to 37	33 to 95	18	3,516
50%	372 to 378	87 to 98	99 to 167	106	17,743
75%	392 to 393	156 to 176	186 to 216	1,189	36,090
100%	798 to 799	507 to 551	401 to 440	31,680	131,627
Time cx. $K=2$	$\sim$ 50 min.	$\sim$ 4 min.	$\sim$ 6 sec.	$\sim$ 1 min.	$\sim$ 8 min.
$K=6$	$\sim$ 1 hour	$\sim$ 5 min.	$\sim$ 7 sec.	$\sim$ 1 min.	$\sim$ 10 min.

Time complexity, on an average laptop, to run the algorithm over various datasets of different sizes where n (respectively $m+1$ and d) is the number of mutations (respectively number of time points and read depth).

References

[1] Laure Barbé, Julien Schaeffer, Alban Besnard, Sarah Jousse, Sébastien Wurtzer, Laurent Moulin, Françoise S Le Guyader, Marion Desdouits, Jean-Luc Bailly, Christophe Gantzer, et al. SARS-CoV-2 whole-genome sequencing using Oxford nanopore technology for variant monitoring in wastewaters. Frontiers in Microbiology, 13:889811, 2022.

[2] Katharina Jahn, David Dreifuss, Ivan Topolsky, Anina Kull, Pravin Ganesanandamoorthy, Xavier Fernandez-Cassi, Carola Bänziger, Alexander J Devaux, Elyse Stachler, Lea Caduff, et al. Early detection and surveillance of SARS-CoV-2 genomic variants in wastewater using COJAC. Nature Microbiology, 7(8):1151–1160, 2022.
