# Supplementary material for: Unsupervised detection and fitness estimation of emerging SARS-CoV-2 variants: Application to wastewater samples (ANRS0160)
Source: PLoS Comput Biol. 2025 Dec 3;21(12):e1013749. doi: 10.1371/journal.pcbi.1013749 (PMC12694877; doi:10.1371/journal.pcbi.1013749)
Supplement: S2 Text — (PDF) [file pcbi.1013749.s002.pdf]

Supporting Information S2 Text
Model assessment over simulated datasets

We started the assessment of our model in the number of groups selection and parameter estimation over various datasets simulated with the same model. Read depths per position and time points are simulated with a Poisson distribution of parameter denoted λ. For clarity, quantities and parameters set in each simulation scheme are summarized in Table S2-1 of the current file where the terme "vary" means that we used various values for the quantity or the parameter in column.

Table S2-1. Summary of simulation schemes.

Scheme name	n	K	$\mathcal{T}$	π	μ	s	λ
Selection$-K1$	100	1	$(0, 5, 12, 20)$	**vary**	-1.5	**vary**	40
Selection$-K3$	100	3	$(0, 5, 12, 20)$	**vary**	(1.5, -1, -2.5)	(-0.1, 0.05, 0.1)	40
Estim$-n-$vary	**vary**	2	$(0, 5, 12, 20)$	(0.6, 0.3, 0.1)	(0.5, -3.0)	(-0.05, 0.10)	40
Estim$-\mathcal{T}-$vary	200	2	**vary**	(0.6, 0.3, 0.1)	(0.5, -3.0)	(-0.05, 0.10)	40
Estim$-\lambda-$vary	200	2	$(0, 5, 12, 20)$	(0.6, 0.3, 0.1)	(0.5, -3.0)	(-0.05, 0.10)	**vary**

Summary of quantities and parameters set for simulation schemes named in the header column. In all simulation schemes, parameters α and β are set to 10 and 50 respectively.

Selection of the number of groups. In order to evaluate the impact of the strength of the signal, in terms of the range of parameter values, on our estimates, we simulated a collection of datasets setting $K = 1$ non-neutral group (simulation scheme Selection$-K1$) or $K = 3$ non-neutral groups (simulation scheme Selection$-K3$) with quantities and parameters listed in the first two lines of Table S2-1. We tested, in particular, the extent to which a selection coefficient or a group proportion decreasing towards zero could impact the selected number of groups. In simulation scheme Selection$-K1$, we used various selection coefficients ($s = 0.025$, $s = 0.050$, $s = 0.075$, $s = 0.100$) and two different vectors of group proportions $\pi = (0.8, 0.2)$ and $\pi = (0.95, 0.05)$. In simulation scheme Selection$-K3$, we used various vectors of group proportions ($\pi = (0.4, 0.2, 0.2, 0.2)$, $\pi = (0.6, 0.2, 0.1, 0.1)$ and $\pi = (0.8, 0.08, 0.06, 0.06)$). We performed 100 replications for each simulation scheme and reported the proportion of models associated with minimal BIC and ICL criteria in Table S2-2.

In this example and as expected, we can see that minimizing a Bayesian criterion for selecting the number of non-neutral groups is highly dependent on the strength of the signal (s in this example) and on group proportions π. As expected, the higher the selection coefficient in Selection-$K1$, the better the model performance in selecting the true number of non-neutral groups. Moreover as one or more group proportion(s) tend(s) towards zero (Selection-$K1$ and Selection-$K3$), the model performances are declining in determining the number of groups and tends to gather groups together. In particular, in simulation scheme Selection-$K3$ (Table S2-2b), group G_2 and group G_3 are the two most similar ones with a negative intercept and a positive selection coefficient. As both their proportion decrease, the model tends to fuse them into one group resulting in a rising proportion of models containing 2 non-neutral groups selected. Moreover, in each simulation scheme, the ICL tends to select a lower number of groups than the BIC which is consistent with the fact that the ICL adds a penalty according to clustering entropy.

Table S2-2. Selection of the number of groups in simulation studies.

		BIC				ICL			
	Number of groups	0	**1**	2	3	0	**1**	2	3
$\pi = (0.80, 0.20)$	$s = 0.025$	29	**71**	0	0	**98**	2	0	0
	$s = 0.050$	0	**100**	0	0	16	**84**	0	0
	$s = 0.075$	0	**100**	0	0	0	**100**	0	0
	$s = 0.100$	0	**99**	1	0	0	**100**	0	0
	$s = 0.500$	0	**100**	0	0	0	**100**	0	0
$\pi = (0.95, 0.05)$	$s = 0.025$	**100**	0	0	0	**100**	0	0	0
	$s = 0.050$	**75**	24	1	0	**91**	9	0	0
	$s = 0.075$	30	**67**	2	1	38	**62**	0	0
	$s = 0.100$	6	**69**	25	0	15	**68**	17	0
	$s = 0.500$	0	**95**	4	1	1	**95**	4	0

(a) Simulation scheme `Selection-`$K1$ with varying s (in rows) and π (one per block of rows).

	BIC						ICL					
Number of groups	0	1	2	**3**	4	5	0	1	2	**3**	4	5
$\pi = (0.80, 0.08, 0.06, 0.06)$	0	5	**60**	32	3	0	0	12	**73**	15	0	0
$\pi = (0.60, 0.20, 0.10, 0.10)$	0	1	20	**79**	0	0	0	1	**52**	47	0	0
$\pi = (0.40, 0.20, 0.20, 0.20)$	0	0	0	**95**	4	1	0	0	2	**94**	3	1

(b) Simulation scheme `Selection-`$K3$ with varying π (in rows).

Proportion of the number of non-neutral groups associated with the likelihood that minimizes the BIC (left) and ICL (right) criteria in simulation scheme `Selection-`$K1$ (Table S2-2a) and `Selection-`$K3$ (Table S2-2b) using various selection coefficients s and/or group proportions π and fixed parameters and quantities listed in the first two rows of Table S2-1. The true number of groups (used for simulations) is in bold in header rows, so is the highest computed value per row.

Parameter estimation and posterior group assignment. In this paragraph, we assume that a number of groups has been estimated as illustrated in the previous paragraph and we assess the performances of our model in parameter estimation and posterior group assignment conditional on a fixed number of groups. We intended to study to which extent our model performances are affected by the number of observations. Label switching, meaning the fact that posterior maxima is invariant to switching labels among non-neutral groups, was handled using the minimum of the mean square error between true (used for simulations) and estimated parameters μ and s where s is multiplied by T in order to obtain quantities of similar range. We excluded π in this mean square error for it to be of very different range.

We used various simulated datasets with quantities and parameter values listed in Table S2-1. In simulation scheme `Estim-n-vary` (respectively `Estim-`$\mathcal{T}$`-vary` and `Estim-`λ`-vary`), we used various numbers of mutations ($n = 25$, $n = 50$, $n = 100$, $n = 200$, $n = 400$) (respectively various numbers of times points ($\mathcal{T} = (0, 5)$, $\mathcal{T} = (0, 5, 9)$, $\mathcal{T} = (0, 5, 9, 12)$, $\mathcal{T} = (0, 5, 9, 12, 20)$) and various Poisson parameter values for sampling read depths ($\lambda = 10$, $\lambda = 25$, $\lambda = 100$, $\lambda = 200$, $\lambda = 400$). We performed 200 replications for each simulation scheme and reported boxplots of parameter estimates in Fig S2-1 (respectively Fig S2-2 and Fig S2-3) for simulation scheme `Estim-n-vary` (respectively `Estim-`$\mathcal{T}$`-vary` and `Estim-`λ`-vary`) as well as boxplots of the Area Under the ROC Curve (AUC) of posterior group assignment in Fig S2-4. AUC were computed using, for each mutation, its true assignment (assigned at simulation) as response and its vector of posterior group assignment, that is

$\left(\mathbb{P}(Z_i = k|X_i = x_i)\right)_{k=0,\ldots,K}$ as predictor.

As expected, as the number of observation increases (increasing n, T or λ), interquartile ranges of boxplots of parameter estimates shrink in expected proportions (Fig S2-1, Fig S2-2 and Fig S2-3). We can also note that, the greater the proportion of a group, the narrower the interquartile ranges associated to its parameter estimates. The parameter estimator being the maximum likelihood estimator, its bias with a limited number of observations n is corrected with an increasing number of observations and becomes negligible from $n = 100$ mutations in simulation scheme `Estim-n-vary`.

Moreover, as the number of observations increases (increasing n, T or λ), AUCs, as expected, tend toward 1 (Fig S2-4). We can also note the very accurate posterior group assignment with AUCs first quartiles above 0.95 in each simulation scheme except for limited read depth ($\lambda = 10$, bottom of Fig S2-4). This result comforted our choice of using the MAP of group assignment for assigning a group to a mutation as well as applying a threshold for read depths during the preparation of WWTP datasets.

Fig S2-1. Boxplots of parameter estimates stratified on the number of mutations n.

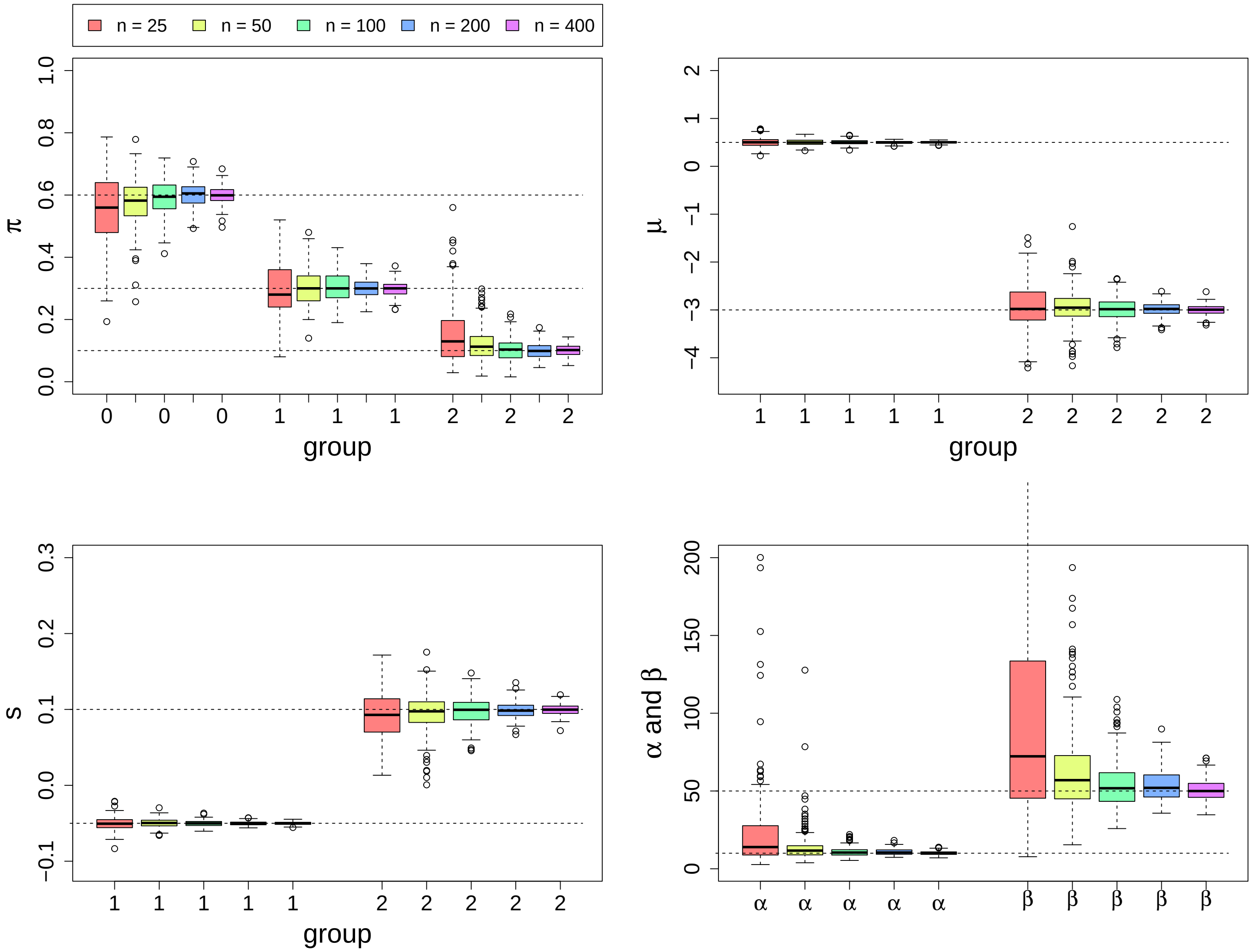

Parameter estimates were computed over 200 replications of simulation scheme `Estim-n-vary` where fixed quantities and parameters are listed in Table S2-1 of the current file. True parameters (used for simulations) are highlighted with horizontal dashed lines.

Fig S2-2. Boxplots of parameter estimates stratified on the number of time points picked from the first to the $(m+1)^{\text{th}}$ value of $\mathcal{T} = (0, 5, 9, 12, 20)$.

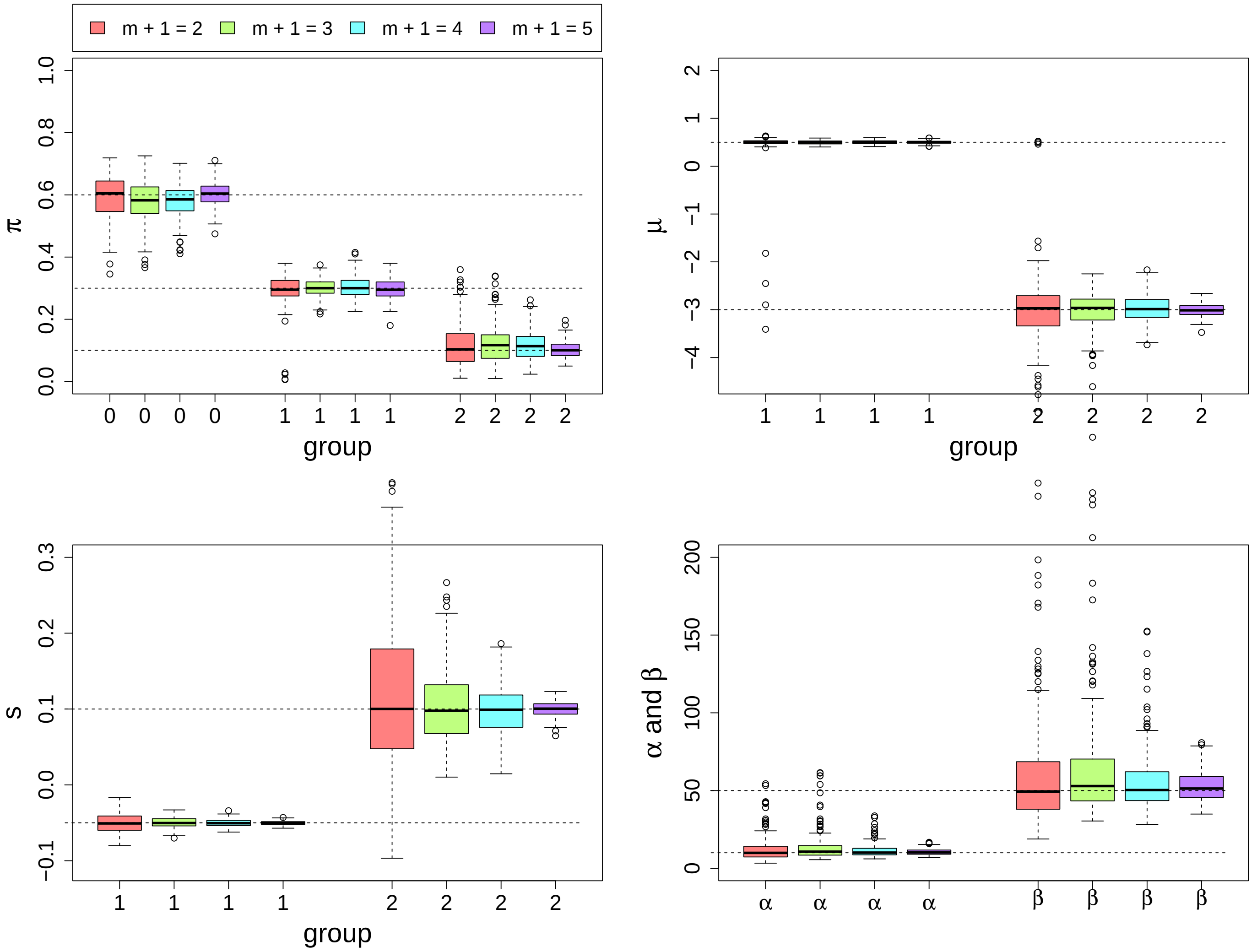

Parameter estimates were computed over 200 replications of simulation scheme `Estim-`$\mathcal{T}$`-vary` where fixed quantities and parameters are listed in Table S2-1 of the current file. True parameters (used for simulations) are highlighted with horizontal dashed lines.

Fig S2-3. Boxplots of parameter estimates stratified on the Poisson parameter λ for the distribution of read depths.

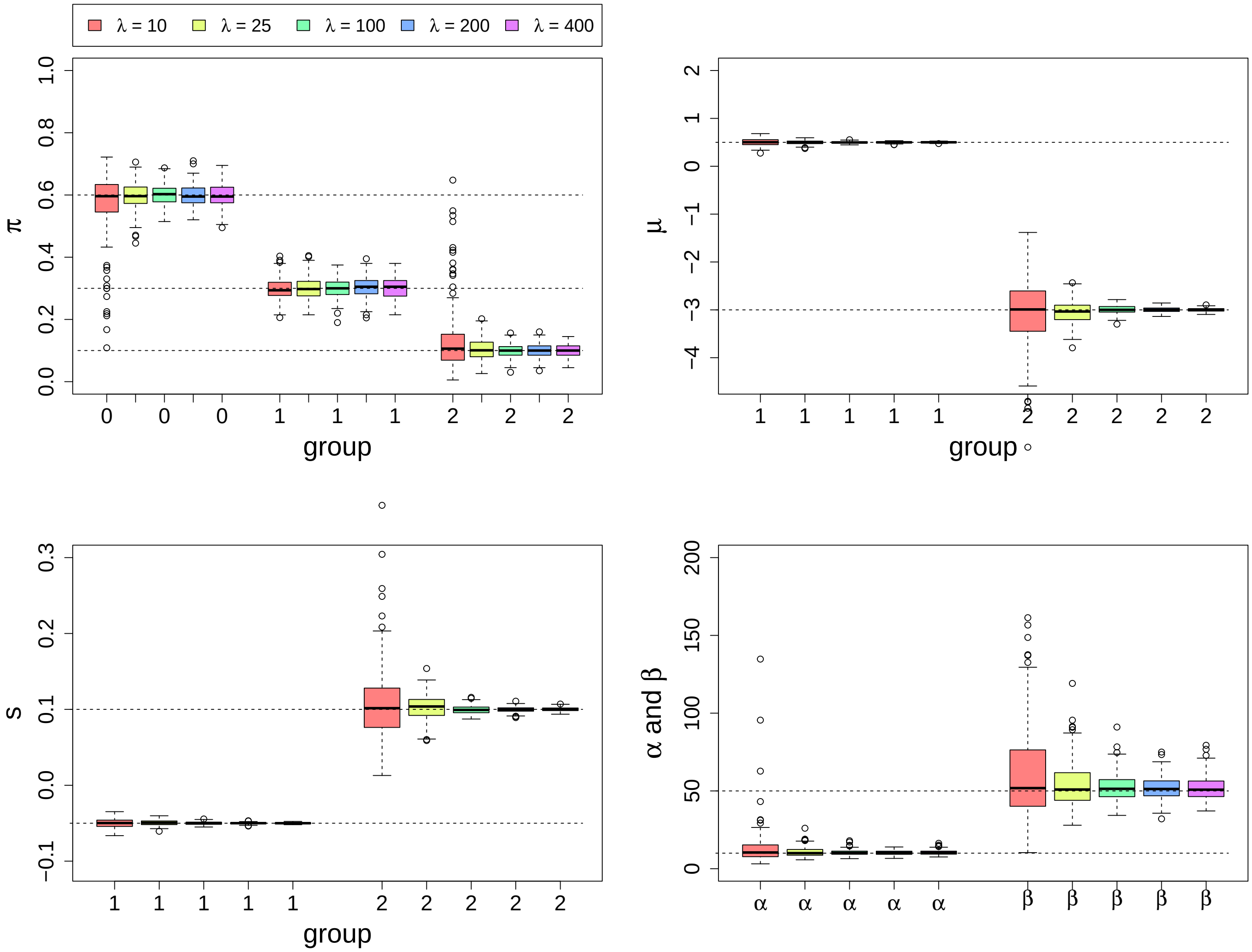

Parameter estimates were computed over 200 replications of simulation scheme `Estim-λ-vary` where fixed quantities and parameters are listed in Table S2-1 of the current file. True parameters (used for simulations) are highlighted with horizontal dashed lines.

Fig S2-4. Boxplots of Area Under the ROC Curve (AUC) of posterior group affectations.

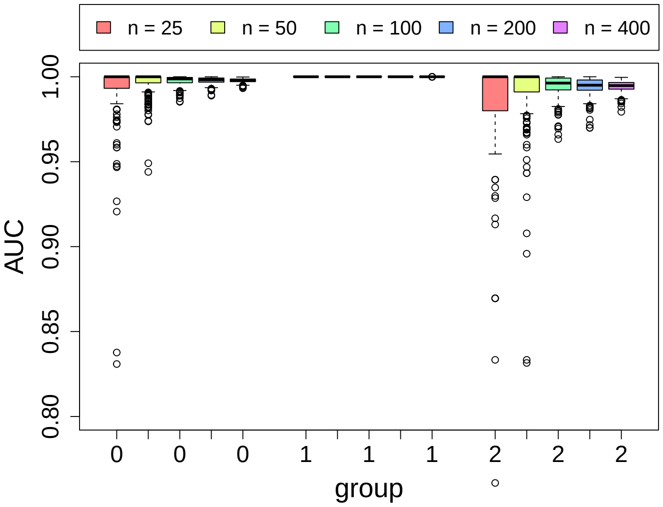

(a) n varies

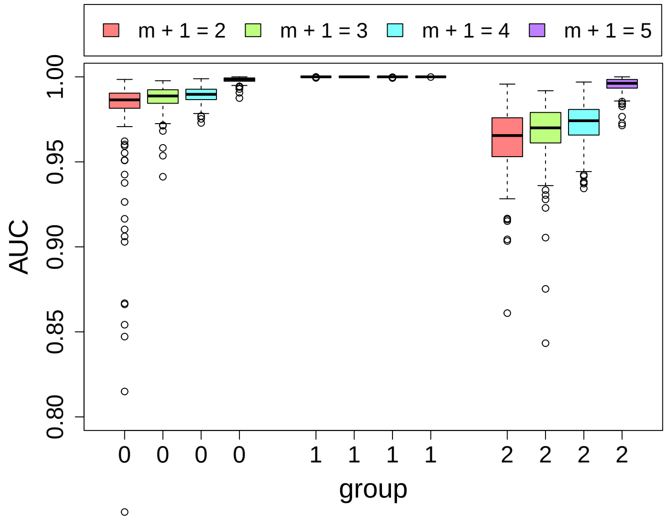

(b) number of time points varies

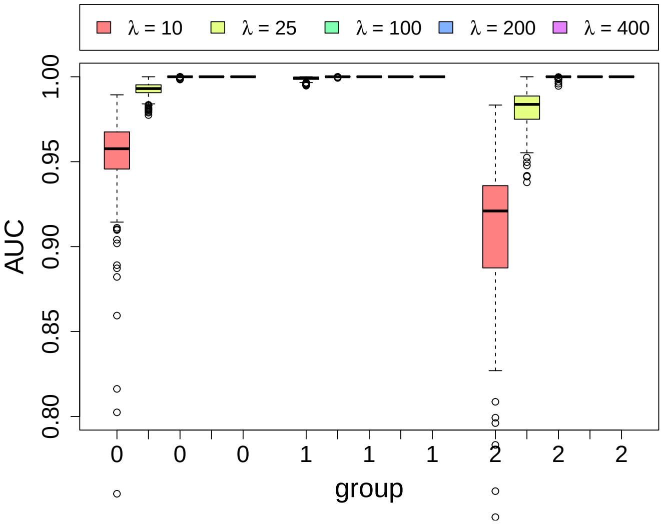

(c) λ varies

Posterior group affectations are those associated to results presented in Fig S2-1 (respectively Fig S2-2 and Fig S2-3) of the current file and stratified on the value of n (respectively $m + 1$ and λ) in top left (respectively top right and bottom) panel.
