# Supplementary material for: Unsupervised detection and fitness estimation of emerging SARS-CoV-2 variants: Application to wastewater samples (ANRS0160)
Source: PLoS Comput Biol. 2025 Dec 3;21(12):e1013749. doi: 10.1371/journal.pcbi.1013749 (PMC12694877; doi:10.1371/journal.pcbi.1013749)
Supplement: S3 Text — (PDF) [file pcbi.1013749.s003.pdf]

Supporting Information S3 Text

The hidden random walk model

Description

Using similar notation as in Section *Method / Mathematical modeling* of the main manuscript, we consider sequencing data collected at $m+1$ increasing time points $t_0, \ldots, t_m$. For $j \in \{1, \ldots, m\}$, let $\Delta_j = t_j - t_{j-1}$ be the difference, usually in days, between time t_{j-1} and time t_j. Let $X_{i,j}$ be the mutation number $i \in \{1, \ldots, n\}$ count at time $t_j \in \{t_0, \ldots, t_m\}$ and $d_{i,j}$ be the read depth at related position at time $t_j \in \{t_0, \ldots, t_m\}$. For $i \in \{1, \ldots, n\}$ and $j \in \{0, \ldots, m\}$, let $Y_{i,j}$ be the logit of the probability parameter of the binomial distribution followed by $X_{i,j}$, the hidden random walk model writes:

$$\mathbb{P}(X, Y, Z|\theta) = \prod_{i=1}^{n} \mathbb{P}(Z_i|\pi)\mathbb{P}(Y_{i,0}|Z_i; \alpha, \beta, \mu, \sigma_0^2)\times \prod_{j=1}^{m} \mathbb{P}(Y_{i,j}|Y_{i,j-1}, Z_i; s, \sigma^2) \prod_{j=0}^{m} \mathbb{P}(X_{i,j}|Y_{i,j}) \quad (1)$$

with

$$\mathbb{P}(Z_i = k) = \pi_k \qquad \text{such that} \qquad \sum_{k=0}^{K} \pi_k = 1,$$

$$\{Y_{i,0} \,|\, Z_i = 0\} = \log\left(u/(1-u)\right) \qquad \text{such that} \qquad u \sim \text{Beta}(\alpha, \beta),$$

$$\{Y_{i,0} \,|\, Z_i = k; k \neq 0\} \sim \mathcal{N}\left(\mu_k, \sigma_0^2\right),$$

for $k \in \{0, \ldots, K\}$, for $j \in \{1, \ldots, m\}$,

$$\{Y_{i,j} \,|\, Y_{i,j-1} = y, Z_i = k\} \sim \mathcal{N}\left(y + s_k\Delta_j, \sigma^2\Delta_j\right)$$

for $j \in \{0, \ldots, m\}$,

$$\{X_{i,j} \,|\, Y_{i,j} = y\} \sim \text{Binomial}\left(d_{i,j}, \text{Logistic}(y)\right),$$

where the Gaussian distribution is denoted $\mathcal{N}(.,.)$ and $s_0 = 0$ by convention. We assume that the model is homoscedastic such that the variance of Gaussian transition probabilities σ^2 is shared across all groups $k \in \{0, \ldots, K\}$ and the variance at time origin σ_0^2 is shared across all groups $k \in \{1, \ldots, K\}$. Such a model better captures the dependency structure of our data, however it is computationally intensive to fit. Nevertheless, let us use it for simulating datasets to analyze with our model described in the main manuscript.

Table S3-1. Summary of simulation schemes.

Scheme name	n	K	$\mathcal{T}$	π	μ	s	λ	σ_0^2	σ^2
`Hidden-RW-`σ_0^2`-vary`	200	2	$(0, 5, 12, 20)$	(0.6, 0.3, 0.1)	(0.5, -3.0)	(-0.05, 0.10)	40	**vary**	0
`Hidden-RW-`σ^2`-vary`	200	2	$(0, 5, 12, 20)$	(0.6, 0.3, 0.1)	(0.5, -3.0)	(-0.05, 0.10)	40	0	**vary**

Summary of quantities and parameters set for simulation schemes named in the header column. In all simulation schemes, parameters α and β are set to 10 and 50 respectively.

Simulation studies

We produced 100 simulated datasets using the hidden random walk model described above with the quantities and parameter listed in Table S3-1. In simulation scheme `Hidden-RW-`σ_0^2`-vary`, we fixed $\sigma^2 = 0$ and we tested different values for standard deviation σ_0 ($\sigma_0 = 0.0, \sigma_0 = 0.3, \sigma_0 = 0.6, \sigma_0 = 0.9, \sigma_0 = 1.2$). In simulation scheme `Hidden-RW-`σ^2`-vary`, we fixed $\sigma_0^2 = 0$ and we tested different values for standard deviation σ ($\sigma = 0.00, \sigma = 0.05, \sigma = 0.10, \sigma = 0.15, \sigma = 0.20$). Note the different scale between σ_0 and σ explained by the fact that both quantities are not expressed in the same unit. Indeed σ_0 is without unit and σ^2 is, for instance, in day^{-1} and of similar order of magnitude of selection coefficients.

Boxplots of parameter estimates represented in Fig S3-1 and Fig S3-2 respectively for simulation schemes `Hidden-RW-`σ_0^2`-vary` and `Hidden-RW-`σ^2`-vary`, show that, as σ_0^2 or σ^2 increases, the bias of the estimator of group proportion π and intercept (α, β for the neutral group and μ for non-neutral groups) increase in large proportions. However, the estimation of the vector of selection coefficients s, which is of most interest, seems to be less impacted by the ignorance of the structure dependency of time series with no bias except s_1, particularly for $\sigma = 0.15$ and $\sigma = 0.20$. We can also note a bias for group assignment towards neutrality (biased estimator of π towards increasing π_0 with increased σ_0 and σ) that may be induced by the beta-binomial distribution for the neutral group. Indeed, the only random parameter being the intercept of the neutral group, it tends to absorb part of the variability of the model. Posterior group assignment is also impacted (see boxplots of AUC of posterior group assignment, Fig S3-3) although their first quartiles stay above 0.7 expect for $\sigma_0 = 0.9$, $\sigma_0 = 1.2$ as well as $\sigma = 0.2$ for the neutral group.

These results suggest that the assumption of independence of mutation counts through time conditional on group assignment, induces a bias when estimating most parameters with our model over datasets simulated with the hidden random walk model. The parameter of highest interest, that is the vector of selection coefficients s, seems however less impacted by such assumption.

Fig S3-1. Boxplots of parameter estimates stratified on the value of standard deviation σ_0.

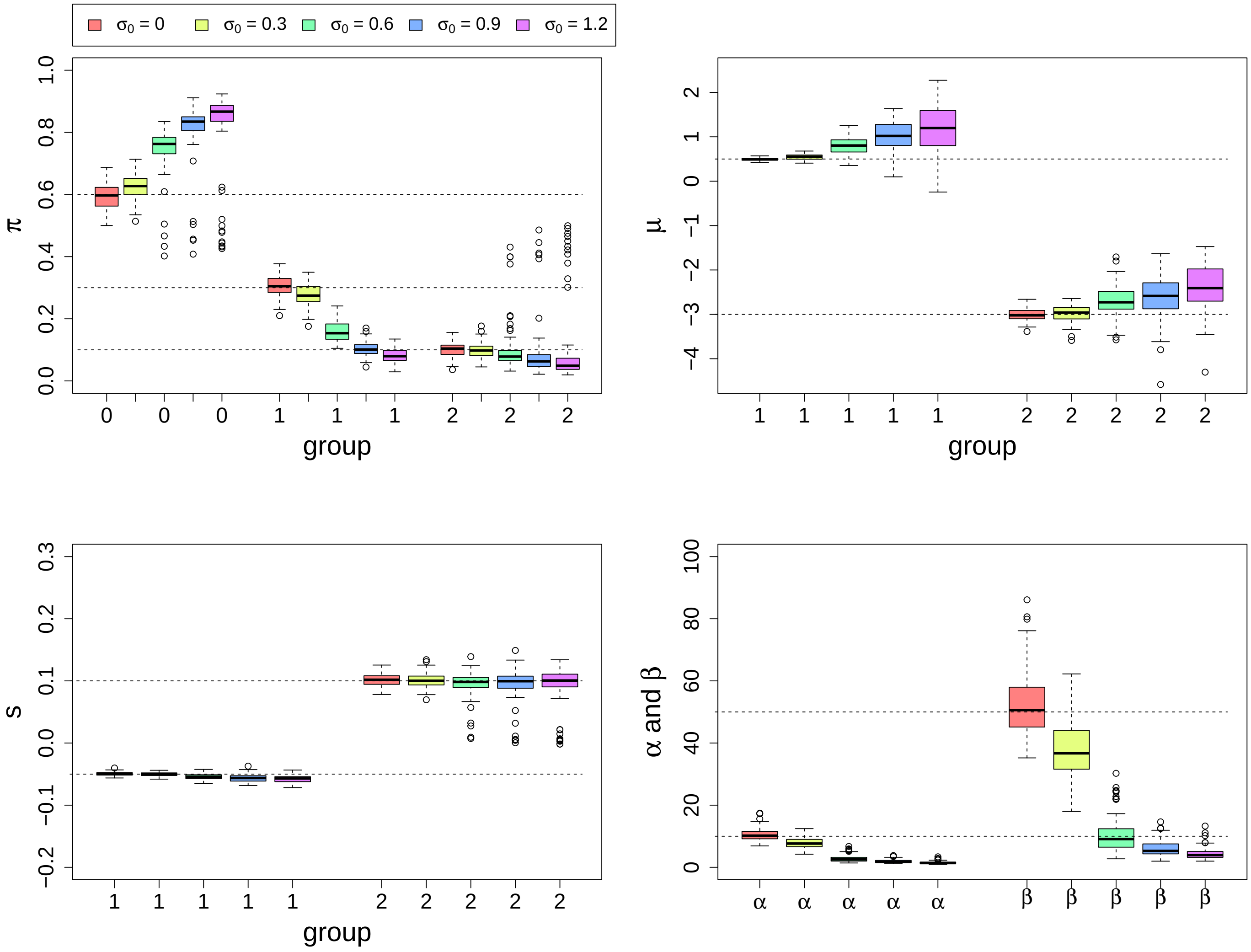

Parameter estimates were computed over 100 replications of simulation scheme `Cond-RW-`σ_0^2`-vary` with the model composed of a random walk conditional on group affectation. Fixed quantities and parameters are listed in Table S3-1 of the current file. True parameters (used for simulations) are highlighted with horizontal dashed lines. The range of the y-axis at the bottom right panel (estimates of α and β) is divided by 2 compared to the one in Supporting Information S2.

Fig S3-2. Boxplots of parameter estimates stratified on the value of standard deviation σ.

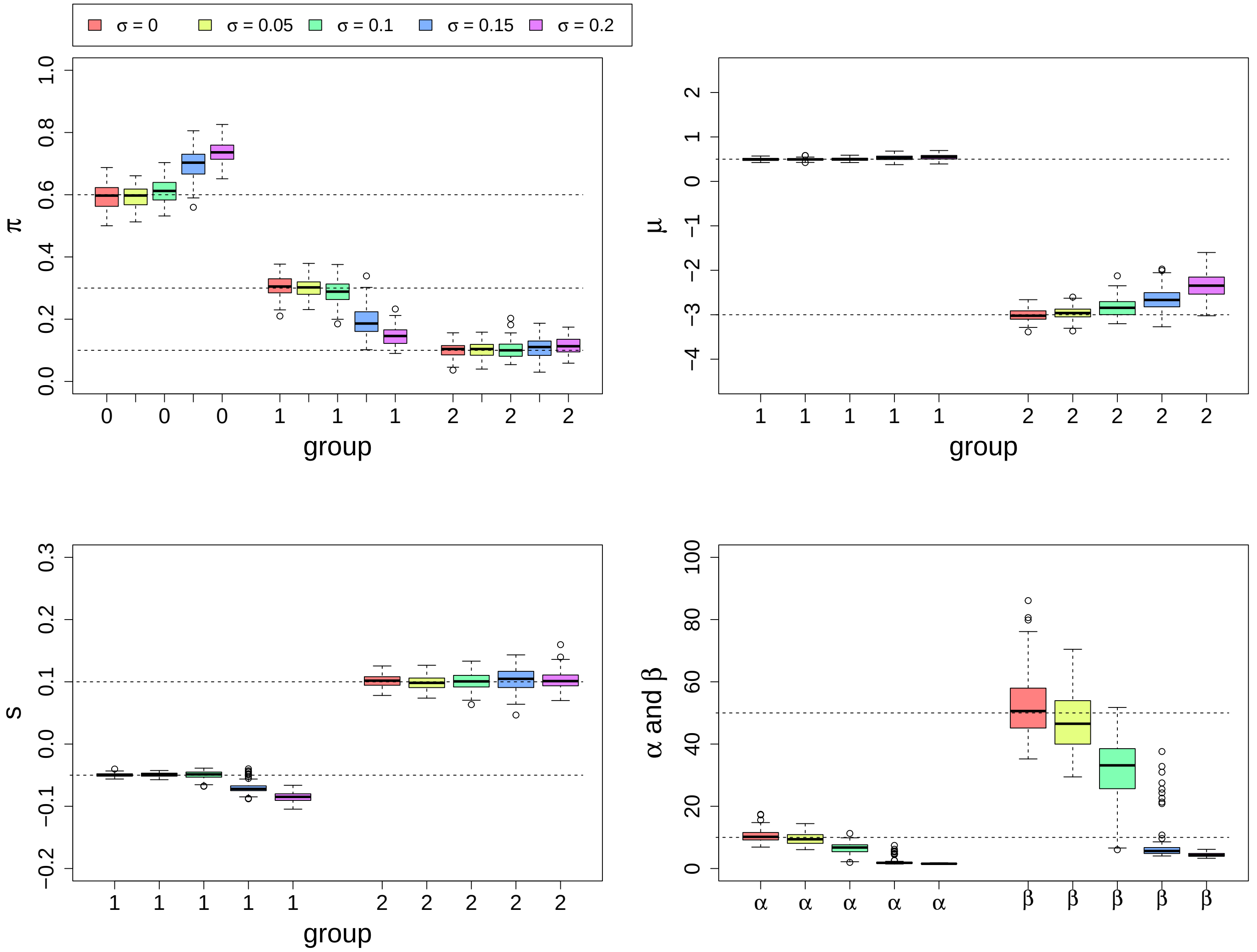

Parameter estimates were computed over 100 replications of simulation scheme `Cond-RW-`σ^2`-vary` with the model composed of a random walk conditional on group affectation. Fixed quantities and parameters are listed in Table S3-1 of the current file. True parameters (used for simulations) are highlighted with horizontal dashed lines.

Fig S3-3. Boxplots of Area Under the ROC Curve (AUC) of posterior group affectations.

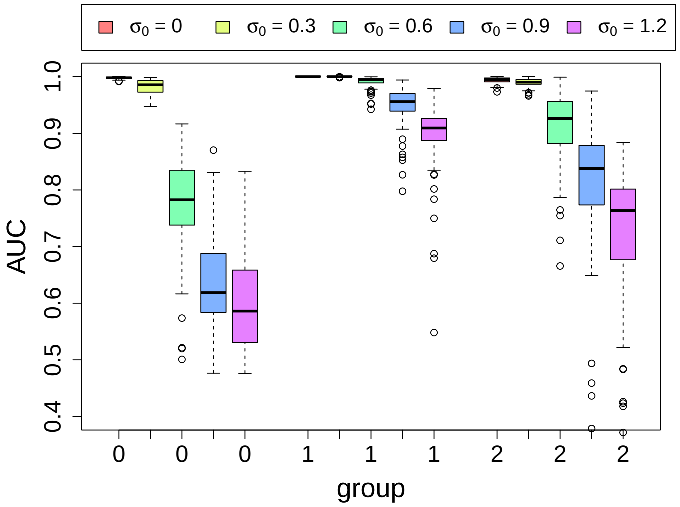

(a) σ_0 varies

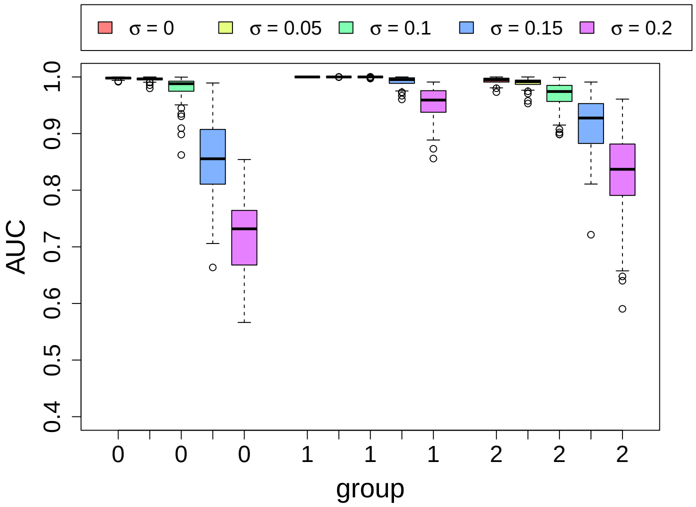

(b) σ varies

Posterior group affectations are those associated to results presented in Fig S3-1 (respectively Fig S3-2) of the current file and stratified respectively on the value of standard deviation σ_0 (left) and σ (right).
