# Supplementary material for: Unsupervised detection and fitness estimation of emerging SARS-CoV-2 variants: Application to wastewater samples (ANRS0160)
Source: PLoS Comput Biol. 2025 Dec 3;21(12):e1013749. doi: 10.1371/journal.pcbi.1013749 (PMC12694877; doi:10.1371/journal.pcbi.1013749)
Supplement: S4 Text — (PDF) [file pcbi.1013749.s004.pdf]

Supporting Information S4 Text

Model entropy

Model (clustering) entropy is given, for a fixed number of groups $K+1$, by $-\sum_{i=1}^{n}\sum_{k=0}^{K}\mathbb{P}(Z_i = k \mid X_i = x_i)\log\mathbb{P}(Z_i = k \mid X_i = x_i)$. Its value reflects to which extent posterior group assignments tend towards zero or one, how well groups are separated. It is quite straightforward to note that the maximal entropy is given by $n\log(K+1)$ (reached for $\mathbb{P}(Z_i = k \mid X_i = x_i) = 1/(K+1)$ for all $i \in \{1,\ldots,n\}$ and all $k \in \{0,\ldots,K\}$). The ratio between clustering entropy and maximal entropy has been computed in analyses `WWTP1-2020-Oct-2021-April` and `WWTP2-2020-Oct-2021-April`. It ranges, for $K = 1$ up to $K = 12$ non-neutral groups, between 0.00 (reached for $K = 1$) and 7×10^{-3} (reached for $K = 7$) in Analysis `WWTP1-2020-Oct-2021-April` and between 4×10^{-9} (reached for $K = 2$) and 6×10^{-3} (reached for $K = 12$) in Analysis `WWTP2-2020-Oct-2021-April`. The very low values of entropy ratio comforted our choice of using the MAP of group assignments for assigning a group to a mutation.
