# Supplementary material for: Unsupervised detection and fitness estimation of emerging SARS-CoV-2 variants: Application to wastewater samples (ANRS0160)
Source: PLoS Comput Biol. 2025 Dec 3;21(12):e1013749. doi: 10.1371/journal.pcbi.1013749 (PMC12694877; doi:10.1371/journal.pcbi.1013749)
Supplement: S5 Text — (PDF) [file pcbi.1013749.s005.pdf]

Supporting Information S5 Text

Additional dataset reduction for analyses covering short time periods

In order to assess the performances of our model in detecting new variants of increased fitness early in time, we performed several analyses over datasets restricted to two times points within the time period of Alpha emergence. For each dataset, mutations of frequency below 0.05 in both samples were removed and read depth below 10 were set to zero along with associated mutation count. An additional dataset reduction was needed in order to remove the extra noise induced by the restriction to two time points. Let us illustrate this issue, for example, over the analysis of WWTP1 dataset restricted to time points 2020-10-20 and 2020-11-04 with the aforementioned dataset reduction.

A set of 93 mutations were assigned to a group of increasing frequency trajectory among which 5 mutations associated to a probability above 0.99 to belong to B.1, B.1.1, B.1.1.7, B.1.160, B.1.177 and/or B.1.351 and 88 mutations associated to a probability below 0.06 to belong to, at least, one of the aforementioned VOC, according to the mutation profile matrix (see Section *Mutation profile matrix* of the main manuscript). Only 6 of these 88 mutations where present after dataset preparation for Analysis `WWTP1-2020-Oct-2021-April` and they were all assigned to the neutral group (see Section *Results / Analyses from October 2020 to April 2021* of the manuscript). The logit of raw frequencies of these 88 mutations is graphically represented in Fig S5 of the current file. Their raw frequencies appear to be of steep increased trajectory between 2020-10-20 and 2020-11-04 which explains their assignment to groups positively selected when restricting the dataset to that time period. They finally show a rapid decrease from 2020-11-04 and an overall near constant trajectory. In order to overcome this issue, we propose an additional reduction strategy with the removal of mutations associated with a probability strictly below 0.005 to belong to B.1, B.1.1 and/or B.1.160, that is main VOC known before the studied time period. In such manner we do not use any information regarding unknown VOC (in particular Alpha) at the beginning of the time period studied (October / November 2020). Moreover, with a threshold as low as 0.005, we keep most mutations of interest and we may remove part of sequencing errors and transient variants.

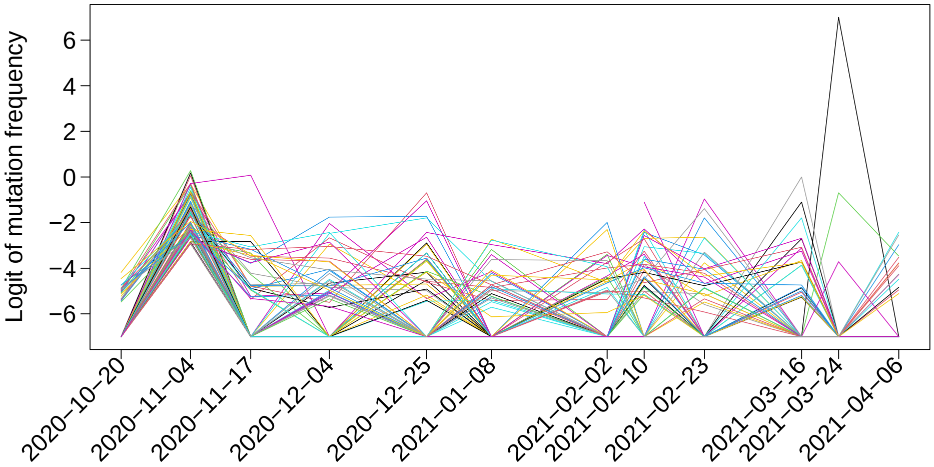

Fig S5. Logit of raw frequencies of the set of mutations assigned a group of positive selection coefficient in the analysis of WWTP1 dataset restricted to time points 2020-10-20 and 2020-11-04 and reduced to mutation of frequency above 0.05 in at least one of the two samples. The set of mutations represented are those associated with a probability below 0.1 to belong to a main VOC and given for the whole time period, from October 2020 to April 2021. Each mutation is associated to one broken line and there is no color code.
