# Supplementary material for: Unsupervised detection and fitness estimation of emerging SARS-CoV-2 variants: Application to wastewater samples (ANRS0160)
Source: PLoS Comput Biol. 2025 Dec 3;21(12):e1013749. doi: 10.1371/journal.pcbi.1013749 (PMC12694877; doi:10.1371/journal.pcbi.1013749)
Supplement: S1 Fig — (PDF) [file pcbi.1013749.s006.pdf]

Supporting Information S1 Fig

	Group									
	0	1	2	3	4	5	6	7	8	9
π	0.64	0.05	0.02	0.06	0.05	0.03	0.03	0.06	0.03	0.03
μ	-1.51	-5.41	-5.63	-2.81	-5.46	-0.40	1.66	-0.90	0.97	1.59
[95%CI]	-	[-5.47; -5.35]	[-5.74; -5.52]	[-2.85; -2.78]	[-5.53; -5.38]	[-0.46; -0.33]	[1.62; 1.71]	[-0.96; -0.85]	[0.93; 1.02]	[1.53; 1.66]
$s \times 100$	0.00	4.67	3.86	2.60	2.40	-1.21	-1.52	-2.03	-2.14	-2.16
[95%CI]	-	[4.62; 4.72]	[3.78; 3.94]	[2.57; 2.63]	[2.34; 2.45]	[-1.29; -1.13]	[-1.57; -1.47]	[-2.17; -1.89]	[-2.19; -2.09]	[-2.23; -2.09]

(a) Parameter estimates.

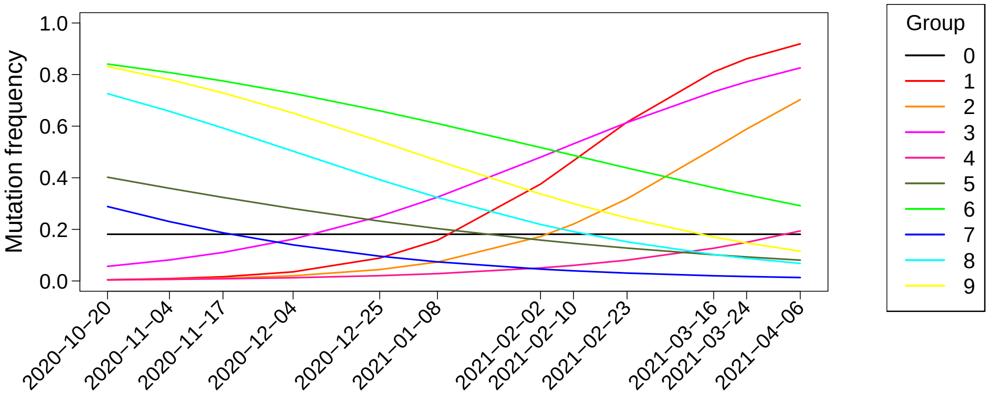

(b) Estimated group frequency trajectories.

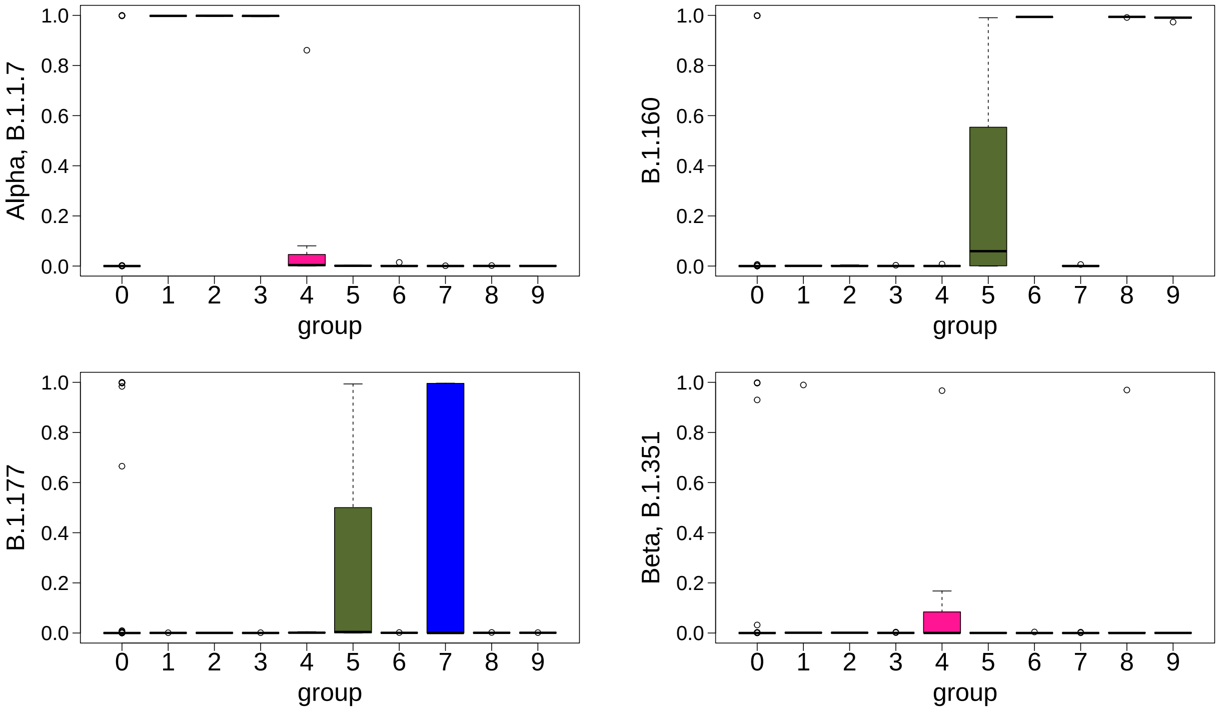

(c) Boxplots of mutation profile stratified on MAP of group assignment.

Analysis `WWTP1-2020-Oct-2021-April` conditional on $K = 9$ non-neutral groups (group size lower limit at 3 mutations). Parameter estimates (a), associated group frequency trajectories (b) and boxplots of mutation profile stratified on MAP of group assignment (c) computed conditional on WWTP1 dataset over all time points (from 2020-10-20 to 2021-04-06) and $K = 9$ non-neutral groups. Selection coefficients and their 95%CI boundaries are multiplied by 100 in (a). The estimate $\hat{\mu}_0$ in (a) is computed from estimates $\hat{\alpha} = 0.53$ and $\hat{\beta} = 2.38$.
