# Supplementary material for: Unsupervised detection and fitness estimation of emerging SARS-CoV-2 variants: Application to wastewater samples (ANRS0160)
Source: PLoS Comput Biol. 2025 Dec 3;21(12):e1013749. doi: 10.1371/journal.pcbi.1013749 (PMC12694877; doi:10.1371/journal.pcbi.1013749)
Supplement: S3 Fig — (PDF) [file pcbi.1013749.s008.pdf]

Supporting Information S3 Fig

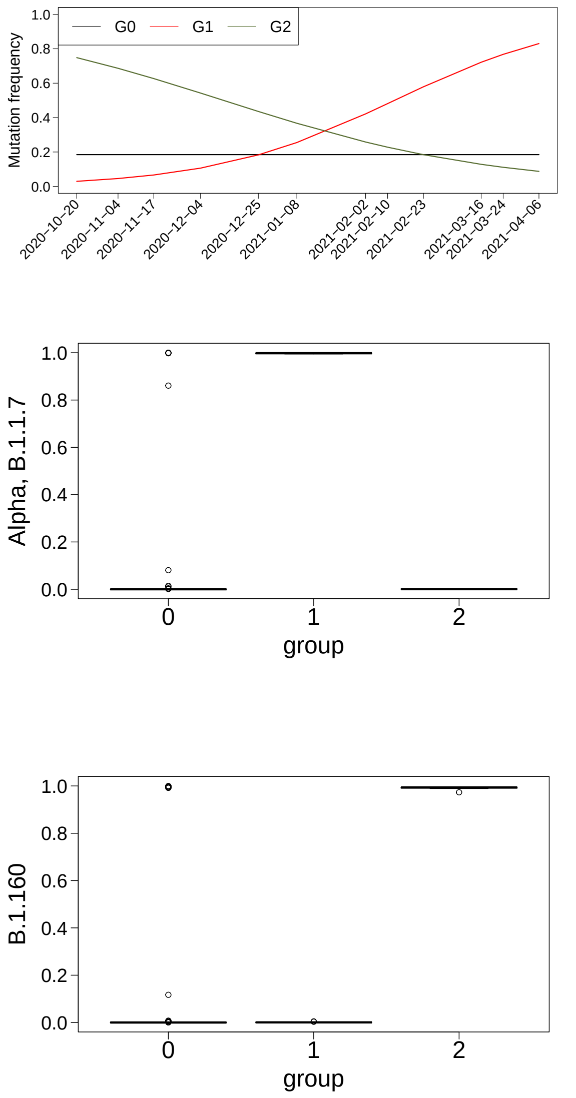

(a) WWTP1 dataset.

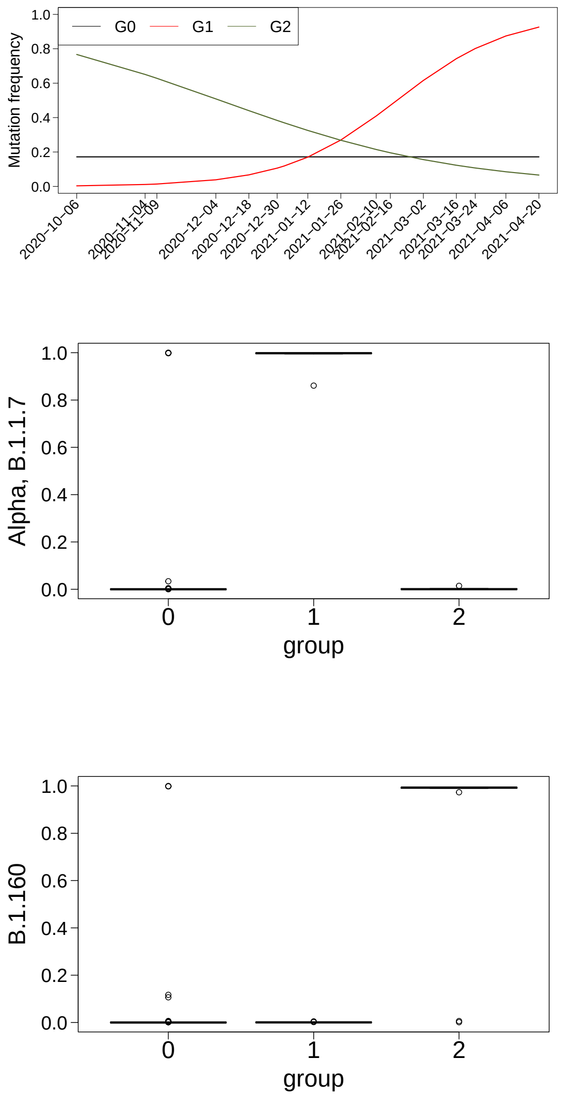

(b) WWTP2 dataset.

Analyses of WWTP1 (left) and WWTP2 (right) datasets over their respective entire period of time conditional on $K = 2$ non neutral-groups.
Group frequency trajectories (left) along with boxplots of mutation profile for B.1.1.7 and B.1.160 stratified on MAP of group assignment (middle and right). Dates 2020-11-08 and 2021-01-02, also composing dataset WWTP2, are removed from x-axis labeling in panel (b, bottom) in order to avoid overlap.
