# Supplementary material for: Unsupervised detection and fitness estimation of emerging SARS-CoV-2 variants: Application to wastewater samples (ANRS0160)
Source: PLoS Comput Biol. 2025 Dec 3;21(12):e1013749. doi: 10.1371/journal.pcbi.1013749 (PMC12694877; doi:10.1371/journal.pcbi.1013749)
Supplement: S4 Fig — (PDF) [file pcbi.1013749.s009.pdf]

Supporting Information S4 Fig

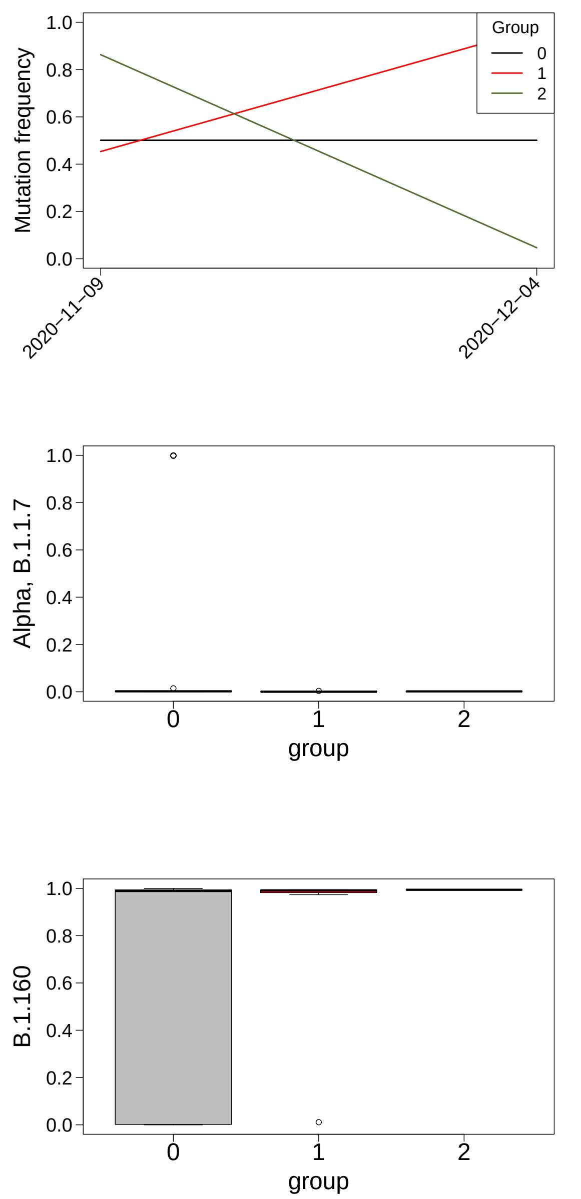

(a) Analysis `WWTP2-2020-Nov-09-Dec-04`.

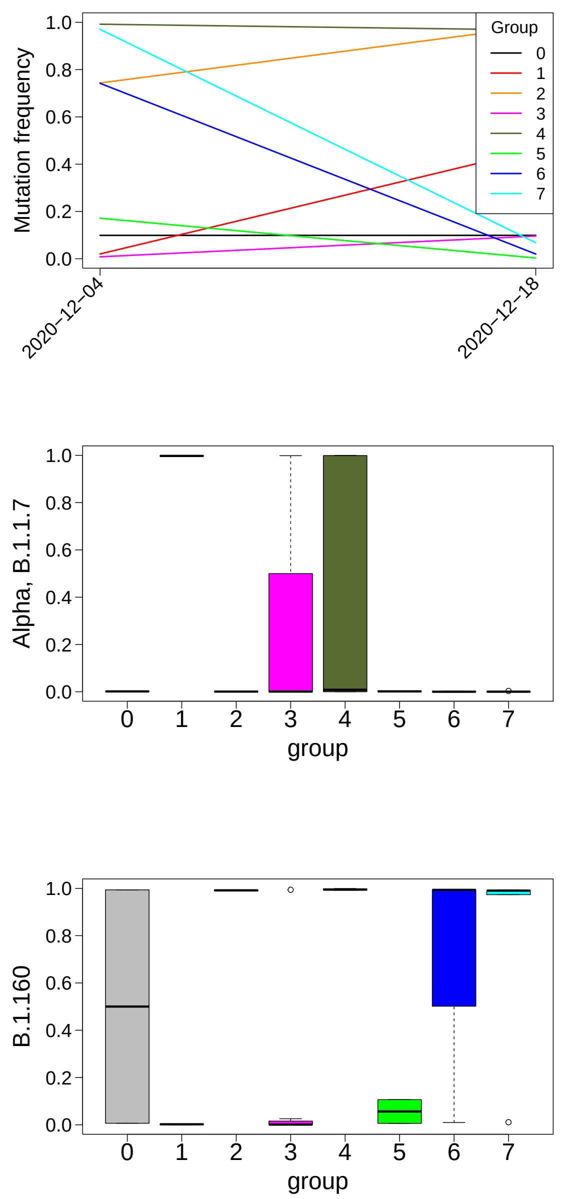

(b) Analysis `WWTP2-2020-Dec-04-Dec-18`.

Analyses of WWTP2 dataset restricted to different time periods between November and December 2020 with a lower group size limit at 2 mutations.
Each panel is associated to one analysis, one period of time. Group frequency trajectories (top) along with boxplots of mutation profile for B.1.1.7 (middle) and B.1.160 (bottom) VOC stratified on MAP of group assignment. The number of non-neutral groups is driven by a lower group size limit at 3 mutations.
