# Supplementary material for: Unsupervised detection and fitness estimation of emerging SARS-CoV-2 variants: Application to wastewater samples (ANRS0160)
Source: PLoS Comput Biol. 2025 Dec 3;21(12):e1013749. doi: 10.1371/journal.pcbi.1013749 (PMC12694877; doi:10.1371/journal.pcbi.1013749)
Supplement: Attachment — Submitted filename: Response to reviewers.pdf [file pcbi.1013749.s010.pdf]

Paris, August 06, 2025

From Alexandra Lefebvre, corresponding author
Postdoc researcher
Laboratoire Jacques-Louis Lions (Sorbonne Université, Paris)
& Centre Interdisciplinaire de Recherche en Biologie (Collège de France, Paris)
Email: alexandra.lefebvre@math.cnrs.fr

To: Andrey Rzhetsky, Academic Editor, PLOS Computational Biology
Roger Kouyos, Section Editor, PLOS Computational Biology

Re: Rebuttal letter to the editor and reviewers who evaluated our manuscript entitled *Unsupervised detection and fitness estimation of emerging SARS-CoV-2 variants. Application to wastewater samples (ANRS0160).*

Dear Editors of *PLOS Computational Biology*,

We would like to deeply thank you and the three reviewers for the evaluation of our work and for inviting us to revise our manuscript. We are very thankful for the constructive suggestions and we feel that answering comments and following suggestions have resulted in a significant improvement of our manuscript.
Enclosed, please find a marked-up copy labeled *Revised Manuscript with Track Changes* and a revised version of our manuscript labeled *Manuscript* entitled

Unsupervised detection and fitness estimation of emerging SARS-CoV-2 variants: Application to wastewater samples (ANRS0160).

In the marked-up copy, significant changes to the manuscript are colored in blue to facilitate identification. Note that additional studies led us to modify the process of dataset preparation. Consequently, the Results section contains numerous modifications although general findings and conclusions stay similar.
Please find below our responses to each point raised by the editor and the reviewers where comments and suggestions are colored in blue.

We greatly appreciate your consideration and remain sincerely,

Alexandra Lefebvre, in the name of the authors.

Journal Requirements

We thank you very much for your careful explanations and guidance for meeting journal requirements. Please find below our answers to your comments :

1) Please ensure that the CRediT author contributions listed for every co-author are completed accurately and in full. At this stage, the following Authors/Authors require contributions: Alexandra Lefebvre, Vincent Maréchal, Obépine Consortium, Arnaud Gloaguen, Amaury Lambert, and Yvon Maday. Please ensure that the full contributions of each author are acknowledged in the "Add/Edit/Remove Authors" section of our submission form. The list of CRediT author contributions may be found here: https://journals.plos.org/ploscompbiol/s/authorship#loc-author-contributions

The CRediT author contributions have been added in the submission form.

2) We ask that a manuscript source file is provided at Revision. Please upload your manuscript file as a .doc, .docx, .rtf or .tex. If you are providing a .tex file, please upload it under the item type u2018LaTeX Source Fileu2019 and leave your .pdf version as the item type u2018Manuscriptu2019.

The manuscript source file is uploaded as a .tex file in the submission form.

3) Please provide an Author Summary. This should appear in your manuscript between the Abstract (if applicable) and the Introduction, and should be 150-200 words long. The aim should be to make your findings accessible to a wide audience that includes both scientists and non-scientists. Sample summaries can be found on our website under Submission Guidelines: https://journals.plos.org/ploscompbiol/s/submission-guidelines#loc-parts-of-a-submission

An Author Summary has been added between the Abstract and the Introduction.

4) We noticed that you used the phrase 'not shown' in the manuscript. We do not allow these references, as the PLOS data access policy requires that all data be either published with the manuscript or made available in a publicly accessible database. Please amend the supplementary material to include the referenced data or remove the references.

All references are now provided either within the text or as Supporting Information.

5) Please upload all main figures as separate Figure files in .tif or .eps format. For more information about how to convert and format your figure files please see our guidelines: https://journals.plos.org/ploscompbiol/s/figures

Figures are uploaded as separate file in .tif format.

6) Please ensure that all Figure files have corresponding citations and legends within the manuscript. Currently, Figures 3, and 4 in your submission file inventory do not have in-text citations. Please include the in-text citations of the figures.

All figures have corresponding citations and legends in the revised manuscript.

7) Thank you for stating "Code for data processing is available at https://github.com/AlexandraLefe." Please provide us with specific link to access the code.

The code is now available at the provided link.

8) Please amend your detailed Financial Disclosure statement. This is published with the article. It must therefore be completed in full sentences and contain the exact wording you wish to be published. 1) State the initials, alongside each funding source, of each author to receive each grant. For example: "This work was supported by the National Institutes of Health (to AM; to CJ) and the National Science Foundation (to AM)." 2) State what role the funders took in the study. If the funders had no role in your study, please state: "The funders had no role in study design, data collection and analysis, decision to publish, or preparation of the manuscript."

Detailed Financial Disclosure statement has been completed according to your requirements.

Answer Reviewer #1's comments

Dear Reviewer,

We thank you very much for your careful review provided in great details. We incorporated your detailed suggestions in the revised version of our manuscript. Changes to our previous manuscript are colored in blue to facilitate identification. Following your main suggestions, we paid a particular attention to the fluidity of the reading, rearranged the Method and the Result sections, moved a significant part of the results as Supporting Information. Note that additional studies led us to modify the process of dataset preparation. Consequently, the Results section contains numerous modifications although general findings and conclusions stay similar. Please find below our answers to your specific comments and suggestions. Your comments are colored in blue in order to enhance the comprehension:

Line 34 – 35: variant classifications are an outcome of the variant's biological characteristics (transmissibility, virulence, immune evasion etc). I think in the first paragraph you should explain why some variants are of concern (e.g., more transmissible, more severe outcomes, or overcoming existing immunity)

Line 5-6: The sentence has been completed in order to specify why some variants are of concern Line 5-6.

Line 41: interest in wastewater genomic surveillance specifically? There is strong interest in using wastewater as an epidemic trend indicator. I think you need to clarify the context of using WW for genomic surveillance of viral evolution, and therefore it is complementing clinical samples (just so readers don't confuse clinical samples as just PCR positivity)

Line 13-15: additional explanations are provided Line 13-15

Line 47: specify here the clinical samples you talk about are individual-specific, in some regions and some phases of the pandemic pooled clinical sampling are used

This is completed Line 18-19

Line 55: why are they inefficient? Is it because we lack information on what specific mutations encode threatening phenotypes? It's ambiguous why these approaches are inefficient.

They are non-parametric such that do not provide parameter estimates. This is completed Line 26-28

Section 2.1: I would like to see a description of the model before the data. In introduction you said you would give general introduction of the model, and apply it to simulations, then finally to real-life data. It is confusing then to see the data presented first.

The description of the model and its assessment over simulated datasets have been moved before the presentation of the data.

Fig 1: the date labels on x axes are not clear – there are overlaps and they are generally small. I suggest stretching out both panels to be wider, and stack them top and bottom, to help visualise the change over time. Also consider adding date ticks. It is a bit difficult to appreciate how many days are in between sampling as you have to mentally calculate rather than read off the graph.

Fig 8 (former Fig 1): Overlapping date labels are removed from x-axis and written in the legend. Both panels are stretched and stack top and bottom. Date ticks are added.

Lines 130-131: is $K = 0$ the neutral group or non-neutral? I am confused, the text here seems to imply it is non-neutral but later on you describe it as if it is the neutral group.

Line 70: G_0 (associated to $Z = 0$) is the neutral group. The sentence has been completed in order to enhance clarity Line 70.

Line 136: note the line number formatting changed in this section. The $X_{i,t}$ (respectively $d_{i,t}$) sentence is confusing – I got it on second read but I think you should just describe the two quantities in separate sentences.

Paragraph following Line 74: There is indeed a line number formatting issue with the lineo package that seems to be not straightforward to solve. We apologize for the inconvenience and we look deeper into the issue.

The two quantities $X_{i,t}$ and $d_{i,t}$ have been introduced separately in order to enhance the comprehension in the paragraph following Line 74.

Top of page 8: The group specific intercepts μ_k are the baseline prevalence of group k at the start of the time series is that right? I think you should explicitly state it to remind readers your groups don't start at the same level (i.e., the observed prevalence is an outcome of the initial condition and relative fitness)

Top of page 6: The intercept μ_k is the logit of the frequency at time origin of group G_k. The precision has been added at the top of page 6 to enhance the comprehension.

Please number your equations for review purposes.

Equations have been numbered

First equation on page 8: you say the selection coefficient makes no distinction between evolutionary or epidemiological parameters. This implicitly assumes the time-varying force of infection contributes to the rate of variant replacement. However your neutral group model has no time-varying component at all – would you expect time-varying FOI to have an effect on random mutation responses too? E.g., if a random mutation happens to have happened as a major epidemic wave begins to rise (but it does not confer an advantage), would you still expect its relative frequency to be still consistent with a time constant beta binomial? I don't have an answer here either but I suspect it might drive your beta-binomial to have a strong degree of overdispersion.

Selection coefficients reflect the overall steepness (or the slope) of the trajectory with no consideration of the causes of the trajectory. Indeed a mutation with an increasing or a decreasing trajectory may be assigned to a non-neutral group, wether it confers a selective advantage or not. This could be the case, for instance, if it belongs to a variant of concern, but also if it rose during a major epidemic wave as a transient variant. This latter issue is highlighted in Table 6 listing the set of mutations assigned to a rising group when analyzing datasets restricted to short periods of time during the emergence of Alpha. We can notice 5 mutations per dataset that were assigned to a rising group although to do not belong to any variant of concern. However, such transient variants are usually assigned to the neutral group when enlarging the time period studied as their overall trajectory remains roughly constant. As our beta-binomiale distribution contains the only random parameter of the model, it tends to absorb the variability of the model.

One line below line 137: wouldn't you have just K pi parameters instead of (K+1) since sum of pi is 1, so you get a free π_0?

Indeed. We added the constrain $\sum_{k=0}^{K} \pi_k = 1$ in the description of the model Line 77.

Line 139: it might be easier to define the binomial rate parameter with a new symbol rather than writing out the logit every time.

Top of page 6: We introduced the notation Logistic for the logisitic function on top of page 6 and we used it in the rest of the manuscript.

Line 144: my experience is that initialisation problem happens with EM/ECM on almost all real data sets. I would frame this paragraph as general approach to the method, not just specifically for the dataset. Related to this, one might also start with quasi-Newton or another faster and less precise method and swap to EM if you are stuck in a flat likelihood region upon initialisation. More generally, does the initialisation trick help with label-switching? I think you need to flag label switching as a common issue for these approaches. You did discuss label switching later in the context of validating against simulated data, but at that point an unfamiliar reader won't know what label switching means.

Supporting Information S2_Text: The initialization procedure has been detailed in Supporting Information S2_Text, extended to a broader framework and complemented with a flowchart for clarity. The model with no neutral group is fast to fit and a quasi-Newton procedure seems to be not necessary for the initialization process. In case of large datasets we made additional suggestions to reduce computational complexity Line 130-135.

Line 178: The notion of label switching has been clarified at Line 178.

Line 163: simulations of synthetic data should be presented in Methods section – it is not obvious why you start results with simulations, or what purpose they serve

Line 141 : Simulation studies have been moved to the Method section under the subsection *Method/Model assessment over simulated datasets* from Line 141

Line 169: this paragraph is narratively awkward – you have not yet introduced the general problem of selecting the numbers of groups, so the simulation reads like a self-contained side exercise rather than a necessary interrogation in the EM latent clustering approach. Again I think you need to move some of the content to Methods to smooth out the narrative. It is also not clear to me why and how you decided on the scheme classifications, since within schemes you have variations anyway. I think you need to start the paragraph by highlighting what model behaviour/biological reality you are trying to tease out with each scheme, then describe their associated parameters. I think this should be in Methods section, with results limited to just how well groups are identified from simulated data.

Line 92: The process of selecting the number of groups has been added and described in the *Method/Clustering and parameter estimation* section (Line 92-108) before the simulation studies.

We added the purpose of our simulation studies at the beginning of related paragraphs (Line 152-154 and 178-179)

Table 2: the results raise a question: does using beta-binomial for neutral group bias group selection towards neutrality, since it would yield different likelihoods than the binomial non-neutral model? I think in principal it makes sense to treat neutral model as beta-binomial but this should be discussed.

Table 3 (former Table 2), Fig 5 and Fig 6: Indeed we would expect a bias in group assignment towards neutrality du to the random parameter of the beta-binomial for the neutral group. However we think this point does not clearly appear in Table 3 (Former Table 2). The tendency of over-selecting zero non-neutral groups in Tables 2a for a decreasing selection coefficient s

could be explained by the fact that, as s decreases, the model tends to estimate its value at zero and fuse the neutral and the non-neutral group. However the bias towards neutrality appears more clearly in an additional result Fig 5 and Fig 6. We report parameter estimates computed over datasets simulated with a hidden random walk conditional on group assignment. We can see that, as the variance of the intercept, or the variance of Gaussian transition probabilities increase (see Line 209-232 for the description of the model), our model tends to overestimate the proportion of the neutral group (Fig 5 and Fig 6).

Line 197: the paragraph on parameter estimation is too verbose and again suffers from being a mix of methods and results

The interpretation of all results have been greatly simplified, repetitions avoided and simulation studies have been moved to the method section.

Figures 2-4: the legends, axes ticks and labels should all be bigger. They are very hard to read in their current form, especially for a complex multi panel figure. Also I suggest using a different style, symbol, background colour etc to differentiate AUC from the rest, since AUC is a performance metric not a parameter so we want to clearly communicate that to readers.

Legends, axes sticks and labels are bigger. The graphical representation of boxplots of AUC have been moved to a different figure.

Line 241: how did you come up with the upper limit for K? From experience I know there is a computational ceiling and higher K would be biologically unreasonable, but you should make it explicit for readers unfamiliar with latent group clustering.

The method section has been completed. The choice of the upper limit for K is detailed Line 108-111.

Line 246: have you introduced entropy in Methods?

Supporting Information S3_Text: A note on entropy model has been added in Supporting Information S3_Text and referenced Line 337.

Figure 5: as expected, BIC and ICL are strongly driven by log L (since parameter space is high and latent mixture models tend to give large absolute values for log L), which raises doubts as to if these criteria are penalising model complexity sufficiently. You can see both criteria appearing to monotonically decrease within your K ranges. This is not reassuring for readers who do not know the caveats about latent mixture models. I feel you need more nuanced text in Methods section to prepare readers for what appears to be poor K selection results at a glance.

Line 92-116: The process of number of group selection has been further detailed Line 92-116 and extended to a broader framework.

Line 258-267: I see you are addressing some of my earlier comments here – these should be moved to Discussion section.

Line 665-676: The discussion regarding number of groups selection has been moved to the Discussion section Line 665-676

Line 268: you already said you were using elbow method in Methods, the justification should be done there.

The process of number of group selection has been clarified and introduced in more details in the Method section

Line 296: again, the text for Methods and Results are blending into one another

Detailed process of dataset analyses has been dispatched in related sections.

Figures 6 to 8: I find the inconsistent labelling between group number and “pi-group number” confusing. In general many of the graphical elements can be tweaked to improve presentation, as suggested in my earlier comments.

Fig 10 and 11 : Group numbers are now labelled with their proper notation G_k. The presentation of the figures has been improved regarding your earlier comments.

Generally, I find labelling analyses with letters confusing. You already have many parameters and model structures for readers to keep track of. I think the analyses should be labelled with more intuitive, if more verbose, names, e.g., WWTP1-20-10-21-04 etc. Better yet, find a way to condense a summary narrative out of the various analyses, or consider only showing a few in the main text and moving the rest to appendices. Overall, the Results section is too verbose. Your average readers will not be interested in all of the different analyses results across time periods, I think much is needed in terms of condensation to strengthen the overall narrative.

Analyses over simulated and wastewater datasets have been labeled with more intuitive names. A substantial part of the analyses are reported in Supporting Information. The Result section has been condensed and clarified. In particular, we avoided to repeat information reported in figures and tables.

Line 615: if the elbow approach worked out well I would say criteria are not the most key limitation. More importantly you could suggest future work to test the robustness of elbow method in K selection. It goes against intuitive to forsake criteria, but if we practically find it working every time then we have confidence in using it.

The method for selection the number of groups has been revised and detailed in the Method section. Moreover future work for empirically testing our method are suggested in the Discussion section.

Line 622: how would you parameterise random effect? I’m assuming it will be time-dependent? I can foresee this would be practically difficult. How about just using beta-binomial for all groups in the future, with shared beta parameters across all groups? That way you enforce higher variability in all groups (which is consistent with overdispersion in the data) without overcomplicating the model. I’m not suggesting you make this change but discuss here as a possible future improvement.

We thought about random parameters for enhancing the variance of the model, for instance, Gaussian parameters. However such model is hard to fit in term of computational complexity as one has to integrate over the random effect during the EM algorithm. We considered beta-binomal for all groups. However, in such models, we do not picture any way of including selection coefficient parameters in order to estimate the strength of the selection. We are currently considering the hidden random walk model described in Equation (3) as a natural future improvement. Such model better captures the structure dependency of the data through time and allows for additional variance through Gaussian distributions for the intercept and transition probabilities.

Line 629: why stop at piecewise regression for selection coefficients, and not take on even more flexible forms like additive splines or Gaussian processes? I think you need to also acknowledge the necessary drawbacks of a more complicated model, and defend the parsimony of your current model as a strength. – Ah I see you immediately discussed this in the following paragraph – I think you could consolidate these paragraphs in one to highlight why you chose to implement your model this way, and its relative merits.

Indeed we could consider more flexible forms of time-dependent selection coefficients. We replaced the term piecewise constant by time-depedent Line 703. Moreover, we added a comment on the advantages of a parsimonious model Line 638-640.

Appendix: for easier use by readers, I suggest uploading your codes in a GitHub repository, instead of being formatted inside a pdf

This has been done in the provided link `https://github.com/AlexandraLefe`.

Also consider reporting computation time - I am very impressed EM in R is even computationally feasible to run for a dataset this size, I imagine many other readers would want to know how scalable this is, especially in comparison to other existing implementations in R through underlying C++ codes.

A paragraph on time complexity has been added Line 117-140. Despite its R implementation, the model fits in less than few minutes in most cases when using parallel computation (and increases with K if one do not parallelize over K.)

We thank you once again for your careful review and your many constructive suggestions and comments. We feel that answering your questions has resulted in a significant improvement to our manuscript.
With our best regards and many thanks,

Alexandra Lefebvre, in the name of the authors.

Answer Reviewer #2's comments

Dear Reviewer,

We greatly appreciate your careful review and your constructive feedback on our manuscript. We have substantially revised our manuscript to incorporate your suggestions. Changes to our previous manuscript are colored in blue to facilitate identification. Note that additional studies led us to modify the process of dataset preparation. Consequently, the Results section contains numerous modifications although general findings and conclusions stay similar. Let us also give brief answers to your comments below. Your comments are colored in blue in order to enhance the comprehension:

Additions to our manuscript due to suggestions by yourself and other reviewers are colored in blue to facilitate identification. Note that additional studies led us to modify the process of dataset preparation. Consequently, the Results section contains numerous modifications although general findings and conclusions stay similar. Let us also give brief answers to your comments below. Your comments are colored in blue in order to enhance the comprehension:

Major Comments:

1. Assumption of Independence in the Joint Probability Expression (Page 8, Line 137): The manuscript appears to assume that observations across different time points are independent. However, real-world time-series data often exhibit clear temporal dependencies. I recommend the authors either provide theoretical justification or include additional simulation studies to examine how violations of this independence assumption could affect the estimation results. Such analysis will significantly enhance the method's practical applicability.

We indeed assume that mutation counts at different time points are independent conditional on group assignment (but not independent without that conditioning). This assumption implies that we ignore the temporal dependency structure of time series. In order to study the impact of such assumption to our results, we conducted an addition simulation study described Line 209-263. We simulated datasets with a hidden random walk model detailed in Equation (3) and performed their analysis with our model detailed in Equation (1). In such manner we studied the extent to wicht an increased variance of intercepts and/or transition probabilities impact our results.

2. Parameter Identifiability: In the proposed likelihood function, there is potential correlation among parameters (e.g., μ and s or others). One challenge in the estimation procedure is ensuring the identifiability of these parameters. I recommend that the authors explicitly discuss parameter identifiability, either theoretically or through targeted simulation studies, to clarify under which conditions these parameters are identifiable.

There may indeed be a potential correlation among parameters. However, we did not empirically encounter any parameter identifiability issue across extensive, various and targeted simulation studies. However, we intend to work on a more theoretical paper that would include the study of the potential correlation among parameters and parameter identifiability in a theoretical way. We also would like to complement our study on time complexity (see Line 117-140) theoretically. We think however that such studies go beyond the current applied work and they

would better fit in a complementary theoretical work. We added a comment in the Discussion section Line 681-686.

3. Effectiveness of the Two-Step Initialization Strategy: The authors propose a two-step initialization strategy to mitigate the sensitivity of the EM algorithm to initial values, which is commendable. However, I would appreciate further discussion regarding its effectiveness with more complex data distributions or larger datasets. Providing insights from the authors' experience on when this initialization strategy is effective and when additional measures might be necessary (without requiring a new explicit strategy) would greatly clarify the generalizability and robustness of the proposed method.

The initialization strategy has been described in greater details in Supporting Information S2_Text. The two step process has been better explained and a flowchart of the process is provided in order to enhance the comprehension.

Minor Comments:

4. Figure 1 Presentation: The current layout of Figure 1, with two subplots placed side by side, appears crowded, and the x-axis dates are overlapping. Additionally, the figure should clearly explain what the different colors represent. At present, the figure does not seem to convey sufficient useful information and may need reformatting for clarity.

Figure 8 (former Figure 1): Subplots have been stacked top to bottom, overlapping dates have been removed from the x-axis labeling and reported in the legend. A comment regarding the fact the there is no color code is more visible, both in the text and in the legend of the figure.

5. Algorithm Pseudocode: To facilitate better understanding and quick comprehension of the proposed algorithm, I suggest including pseudocode or a flowchart in either the main text or the appendix.

Supporting Information S2_Text is complemented with a flowchart.

6. Availability of Code: Currently, the code files are not available through the provided GitHub repository (although some snippets are present in the appendix). To further improve the transparency and reproducibility of this research, I strongly encourage the authors to publicly release the code and provide detailed README documentation, enabling other researchers to easily reproduce the experimental results.

The code for dataset preparation and the code of the algorithm has been made available at the link `https://github.com/AlexandraLefe`.

We thank you once again for your constructive comments. We feel that your suggestions permitted to greatly enhance our manuscript.
With our best regards and many thanks,

Alexandra Lefebvre, in the name of the authors.

Answer Reviewer #3' comments

Dear Reviewer,

We thank you very much for your constructive feedback on our manuscript. Following your suggestions, we have made several changes to the manuscript. Changes to our previous manuscript are colored in blue to facilitate identification. Following your main suggestions, we paid a particular attention to enhance the readability of the manuscript. Note that additional studies led us to modify the process of dataset preparation. Consequently, the Results section contains numerous modifications although general findings and conclusions stay similar. Let us also provide an answer to your specific comments and suggestions below. Your comments are colored in blue in order to enhance the comprehension:

1) Remove any data from the text that is also mentioned in figures or tables. On numerous occasions, the authors present information in the text that is also mentioned in tables. A clear example of this is the second paragraph of the results (line 171 "setting K =1 non-neutral group... " until "in table 2.", line 181). This information is also available in table 1, and even added to table 2, and therefore available to the reader, but makes the paragraph very hard to read. This happens again on page 13, from line 203 onwards, as well as line 331 (page 22), line 407 (page 28), line 422 (page 29), and line 530 (Page 38). There are probably more places where information can be removed from the text and just referred to the table to improved the flow of the text.

Most data or results present in figures and tables have been removed from the text.

2) Don't reference in forward direction. The authors have a tendency to refer to sections of the paper that the reader hasn't read yet. In particular, to section 4 (Discussion and perspectives). This is rather disruptive to the reader, as it feels like from there on, vital information is missing when following the paper chronologically, because the Discussion hasn't been read. Make sure that the presentation of results is done in such a way that it follows logically to the reader. The results section presents the results of the analyses proposed in the methods section, and the reader expects their implications to be discussed in the discussion section. There is certainly room for interpretation in the results section, but if the narrative is consistent, the reader can wait for wider implications to be discussed later on (in the Discussion section).

We removed all references in forward direction.

3) Refer to analyses and sections by name, not by letter. Throughout the paper, different parts of the analysis are referred to by letter (e.g. "Simulation scheme A", "Section 4", "Analysis B and C"). This means that the reader has to go back into the text or tables to find out which parts are referred to. I would want to suggest to refer to all these parts with some name rather than the letters, to make it easier for the reader.

Analysis have been referred with intuitive names. Sections have been referred by names.

4) Move part of the analyses to a supplementary information All in all, the text is rather long, and some of the sub-analyses seem repetitive (but still needed!). Similar simulation schemes could for instance be moved to the supplementary information, with their differences mentioned in the main text. Shortening the text will make it more accessible to a larger audience.

A substantial part of the analyses have been moved to Supporting Information

5) Be specific In the methods section, the authors use “few iterations” twice, and “few time” once. Please be specific on how many times this was done. Currently this reads like “After fiddling around a bit, the initialisation magically worked.”. The same applies to the use of the “Elbow method”: it has no clear criteria for determining where the “elbow” actually is located, making it very subjective. If the method is to be used in real-time to detect variants, specific criteria for the selection of the number of clusters are needed.

The method is described and complemented with greater details.

- Given that the method is first tested on simulated data, I would suggest moving the subsection on Data (2.1) to after the one on Mathematical Modeling (2.2).

Simulation studies have been moved at the end of the Method section.

- Page 6, line 125: the logit transformation of mutation frequencies is not purely practical for visual purposes. The expected trajectory of a fitness-conferring SNP is linear when logit-transformed.

Our comment was solely meant for the graphical representation. We removed it from Line 319-326 in order to avoid any confusion.

- A matter of taste: I would suggest renaming the section “Real data” to Wastewater data”, to avoid any discussion on what “Real” means.

The simulation studies have been moved to the Method section and the section “Real data” has been named “Results”

- figure 6 says “log frequency”, instead of “logit frequency” in the legend for panel (b)

Thank you, this has been changed in Figure 10 (former Figure 6).

- Please note that “public health strategies” (page 26, line 363) are not expected to influence selection, and with that the mutation’s frequency trajectory, because PH strategies generally influence all variants equally in the absence of methods to distinguish between cases caused by different variants in the field in real-time.

The term public health strategies has been removed from Line 473.

We thank you once again for your constructive comments. We feel that your suggestions permitted to greatly enhance our manuscript.
With our best regards and many thanks,

Alexandra Lefebvre, in the name of the authors.
