# Supplementary material for: Unsupervised detection and fitness estimation of emerging SARS-CoV-2 variants: Application to wastewater samples (ANRS0160)
Source: PLoS Comput Biol. 2025 Dec 3;21(12):e1013749. doi: 10.1371/journal.pcbi.1013749 (PMC12694877; doi:10.1371/journal.pcbi.1013749)
Supplement: Attachment — Submitted filename: Response_to_reviewers_auresp_2.pdf [file pcbi.1013749.s011.pdf]

Paris, November 12, 2025

From Alexandra Lefebvre, corresponding author
Postdoctoral researcher
Laboratoire Jacques-Louis Lions (Sorbonne Université, Paris)
& Centre Interdisciplinaire de Recherche en Biologie (Collège de France, Paris)
Email: alexandra.lefebvre@math.cnrs.fr

To: Andrey Rzhetsky, Academic Editor, PLOS Computational Biology
Roger Kouyos, Section Editor, PLOS Computational Biology

Re: Rebuttal letter to the editor and reviewers who evaluated our manuscript entitled *Unsupervised detection and fitness estimation of emerging SARS-CoV-2 variants. Application to wastewater samples (ANRS0160).*

Dear Editors of *PLOS Computational Biology*,

We would like to deeply thank you and the three reviewers once again for the thorough evaluation of our work. The very constructive remarks have permitted to greatly enhance the value of our work both in terms of scientific content and clarity.

We thank again Reviewer #1 for his/her new comments and we feel that following his/her additional suggestions permitted to improve further the clarity of the manuscript. In the marked-up copy of our manuscript, the modifications are colored in blue to facilitate identification.

Please find below our responses to each point raised by the reviewer where comments and suggestions are colored in blue.

We greatly appreciate your consideration and remain sincerely,

Alexandra Lefebvre, in the name of the authors.

Answers to Reviewer #1's comments

Dear Reviewer,

We thank you once again for your careful review and your many constructive suggestions and comments. We are thankful for your additional suggestions and we feel that following them has resulted in a significant improvement in the clarity of our manuscript.

In the marked-up copy of our manuscript, the modifications are colored in blue to facilitate identification. Please find below our answers to your suggestions. Your comments are colored in blue in order to enhance the comprehension:

First, although the rationale of your simulation studies and method for group number selection have been much improved in the main text, you now have a very long Method section, which in itself contains the results from the simulation study. This makes the overall narrative of your paper hard to follow as readers can get lost in the details in the methods. At the same time, the Method section, despite its length, has not sufficiently contextualised the need and the challenge of selecting groups. I propose the following changes: at the end of the 'Mathematical modelling' section, use some brief text to explain why you set up the model the way it is, and prompt the reader that the main implementation challenge is to simultaneously estimate the number of groups and group selection coefficients. Then, I suggest heavy-handed edits to condense the "Clustering and parameter estimation" – keep it short and clear that these are the necessary and complex approaches needed to solve these implementation challenges. I think you will have to move most of the existing text in this section to an appendix, and leave the main text as mostly a summary.

An important rearrangement of Section "Method" has been performed in order to improve the clarity of the manuscript and avoid a dilution of the key message. Names of Section "Clustering and parameter estimation" and paragraph "Statistical tools" have been exchanged for consistency and clarity. Section "Statistical tools" (former Section "Clustering and parameter estimation") has been much condensed in the following way:

- Paragraph "Clustering and parameter estimation" (former "Statistical tools") has been mostly moved to Supporting Information S1 and turned into a brief mention of the main tools we used in the text.
- Paragraph "Selection of the number of groups and posterior group assignment" has been renamed "Selection of the number of groups" for clarity. It begins with a note on the complex task of selecting the number of groups in unsupervised clustering in order to drive the reader towards its motivation and one of the challenges.
- Paragraph "Algorithmic complexity" has been moved to Supporting Information S1 in full as it is of secondary importance.

NB: we do not fully understand the suggestion of brief text to add at the end of Section "Mathematical modeling" though as, to our point of view, parameter estimation + clustering and

the estimation of the number of groups are performed as two separate tasks and we hope the aforementioned changes are sufficient to enhance the clarity of the manuscipt.

Likewise, the 'Model assessment over simulated datasets' section needs to be condensed, and you need to be more explicit about its motivation, i.e., the nature of the model set up makes it challenging to identify group number and selection, so you ran a simulation study as proof of the robustness of your approach. I feel the same way about 'The hidden random walk model' section – it is good that you have explored the consequences of not explicitly encoding temporal correlation structure, but by the time the reader finishes this verbose section, they might become distracted or disoriented about the main message of the overall paper. I strongly recommend condensing the Methods section as much as possible, and especially pay attention not to introduce too many results of the simulation/random walk studies. In my opinion at most you could summarise the results of those complementary studies in text, but you should leave the figures to supplementary materials. You have too many figures as it stands now anyway.

Section "Model assessment over simulated dataset" has been much condensed. It is divided in two parts. The first part is related to simulation studies over datasets simulated with the same model for a global assessment. The second part is related to the hidden random walk model for testing the assumption of conditional independence. Each of these two part starts with its explicit motivation. Only a summary of main results are kept in the manuscript and all figures and details are moved to Supporting Information S2 (respectively Supporting information S3) for the first (respectively the second) part.

There is a degree of dissonance between the Methods and the Results sections, which are both verbose and too long. While the Methods go into great details justifying the robustness of the approach, the Results likewise is very detailed in biological interpretations of the specific datasets, in their unique epidemiological contexts. These results are important, but it is confusing how they echo with the narrative in the Methods. For example, the comparison with supervised results, which in my opinion is crucial to the overall narrative, is not well anticipated in the Methods section. If you had just read the Methods section you do not get the impression that these Results would be reported. I admire the wide array of scientific queries you must have had to address to produce this work, but I worry this breadth is diluting the narrative cohesion of your manuscript, and as a result, is weakening its quality. Although it is clear all parts of the results are laborious to obtain, I think the way forward is to select the key message to present (which in my mind is how the unsupervised classification results compare against supervised ones, and what that tells us about the usefulness of your novel approach), and consolidate everything else into brief summaries, and provide full details in supplementary material.

As a result of your first suggestions, we believe that the aforementioned changes improve also the cohesion of the manuscript and prevent from a dilution of the key message. Moreover we moved forward our intention to compare our results to supervised and retrospective ones at the end of the first paragraph of Section "Results" (lines 173-175) (along with Section "Introduction"). Furthermore Section "Parameter estimation" has been renamed "Clustering and parameter estimation with comparison to supervised and retrospective approaches" in order to anticipate our intention to compare and verify our results. Additionally, in order to lighten Sec-

tion "Results", part of the details regarding dataset preparation for Section "Fitness detection" have been moved to Supporting Information S8.

To summarise, I believe the core scientific merit of this work is demonstrating how an unsupervised classification approach can uniquely provide early genomic shift signals in epidemic surveillance, including whether new strains have begun circulating and whether they have transmission advantage. This new approach is important in prompting timely response. The genomic surveillance over COVID pandemic and the unique epochs dominated by a succession of variants provided a unique data opportunity for the author team to test such an approach. However, implementing an unsupervised classification is hard work, and requires thorough stress-testing of both the model design and the implementation algorithm. I think at the moment the manuscript goes into too much detail about these implementation hurdles and loses the big picture of its impact in an epidemic response context.

We hope the aforementioned changes have sufficiently improve the cohesion of our manuscript with an emphasis on the key motivation and impact of our work and a diminution of the focus on the methodological challenges.

As a side, I think this manuscript has too many figures, and the figures are needlessly complex. For example, the trendlines on Fig 8 are too messy to make sense of, and colour legend is missing – what is important to note on this figure? I do not think the data needs to be visualised just for visualisation sake, if it is so hard to make out a latent pattern from the plot. Likewise figures 10,11,13 have too many panels and the graphical elements are too small to see, you need to adjust line width, legend size etc for all of them. Once again I encourage thinking about which of those visualisations actually help with the narrative and which can be shelved into supp material.

Former Figure 8 has been removed. Figures 2 and 3 (former figures 10 and 11): the panel displaying the logit of group frequency trajectories has been removed. We believe that keeping the graphical representation of group frequency is an important visual support. Graphical elements, line width and legend size have been enlarged. Former figure 13 has been split in two with Figure 5 and Figure 6 and graphical elements have been enlarged.

We thank you for your very careful reviews and your constructive comments. We feel that your additional suggestions permitted to greatly enhance our manuscript once again.

With our best regards and many thanks,

Alexandra Lefebvre, in the name of the authors.

Response to Reviewer #3

Dear Reviewer,

We would like to thank you once again for your previous comments that helped us to considerably enhance the quality of our work and we are very pleased that you are satisfied with the revised version.

With our best regards and many thanks,

Alexandra Lefebvre, in the name of the authors.
